# Supplementary material for: Toward essential oil stewardship: strain-resolved evaluation of thyme oil activity against Pseudomonas aeruginosa
Source: Front Pharmacol. 2025 Sep 26;16:1659096. doi: 10.3389/fphar.2025.1659096 (PMC12510957; doi:10.3389/fphar.2025.1659096)

Supplementary Material

**Supplementary Table S1.** Statistical analysis of the data distribution for biofilm features of separate *P. aeruginosa* strains (n=10). Distribution of biofilm mass **(A)**. Distribution of biofilm metabolic activity **(B)**. Normal distribution was considered for values of p>0.05 (Shapiro-Wilk test). SD- standard deviation, IQR- interquartile range (IQR), N- data points.


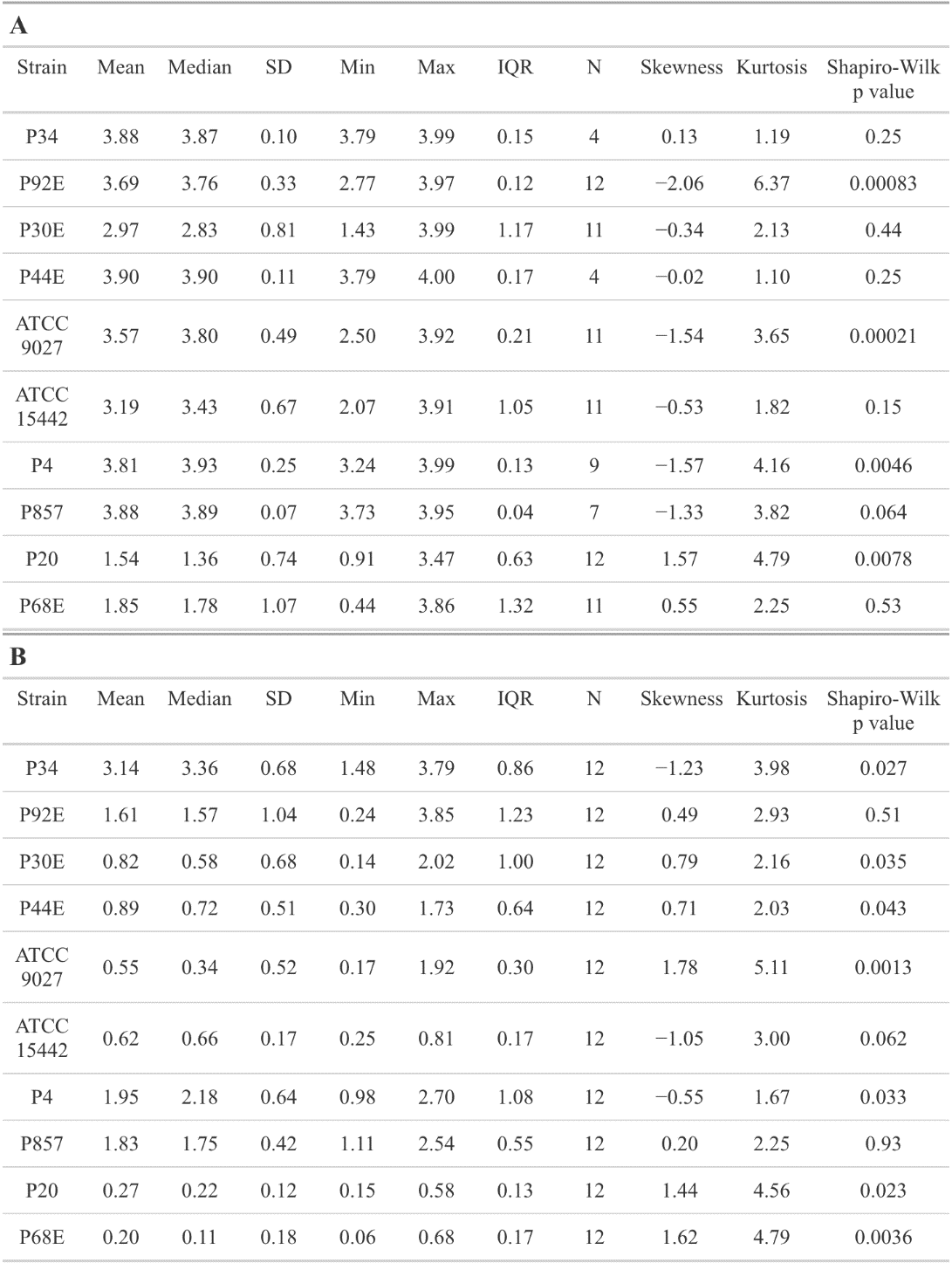


**Supplementary Table S2.** Parameters of the tested statistic of the differences in biological mass between particular *P. aeruginosa* strains (n=10). Welch’s ANOVA, followed by the Games-Howell test, was performed. Values of p<0.05 were considered significant, p<=0.05 was marked with one asterisk, p<=0.01 was marked with two asterisks, p<=0.001 was marked with three asterisks, and p<=0.0001 was marked with four asterisks. Ns- no significant differences, upper-lower Cl- confidence interval 95.


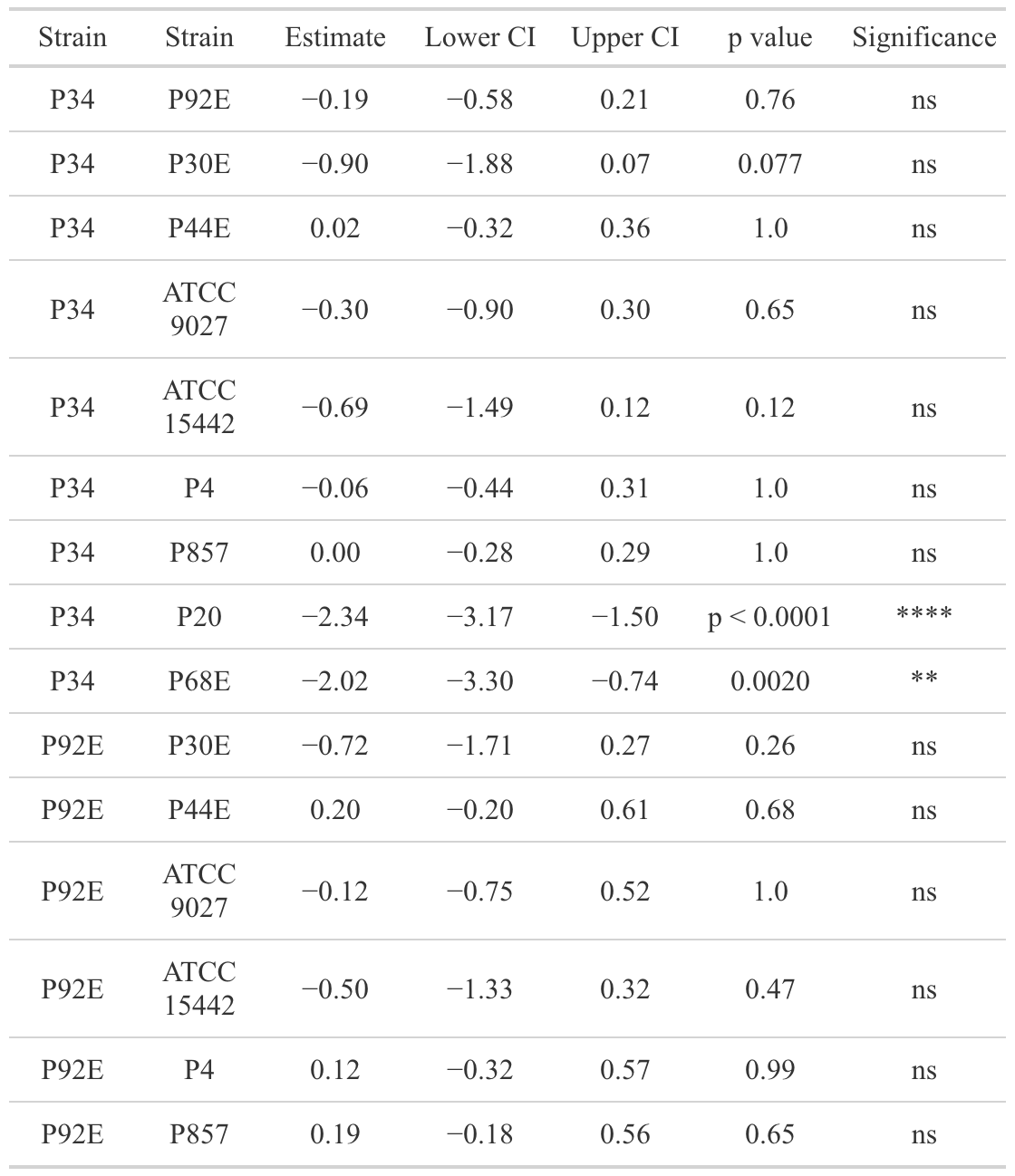


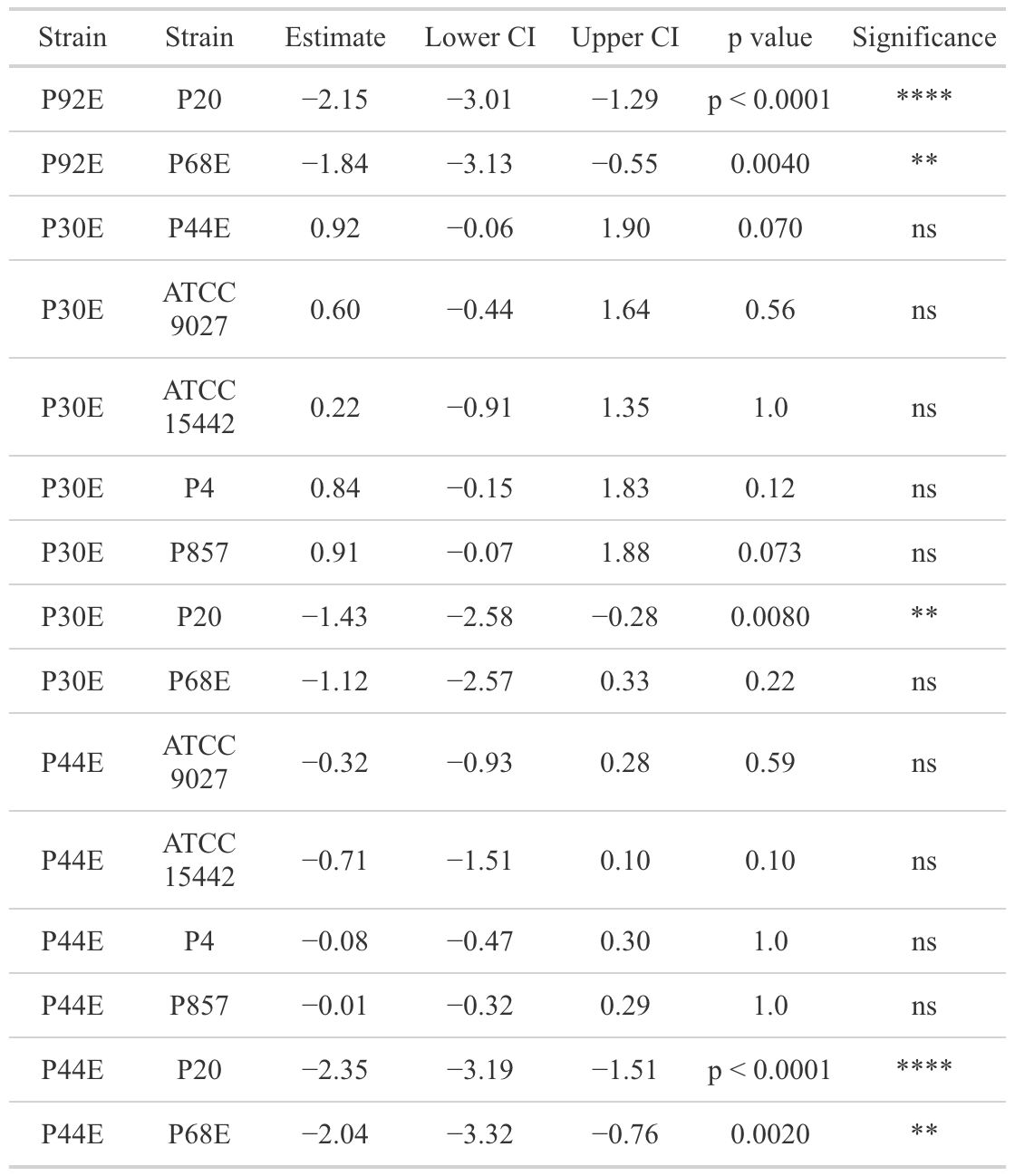


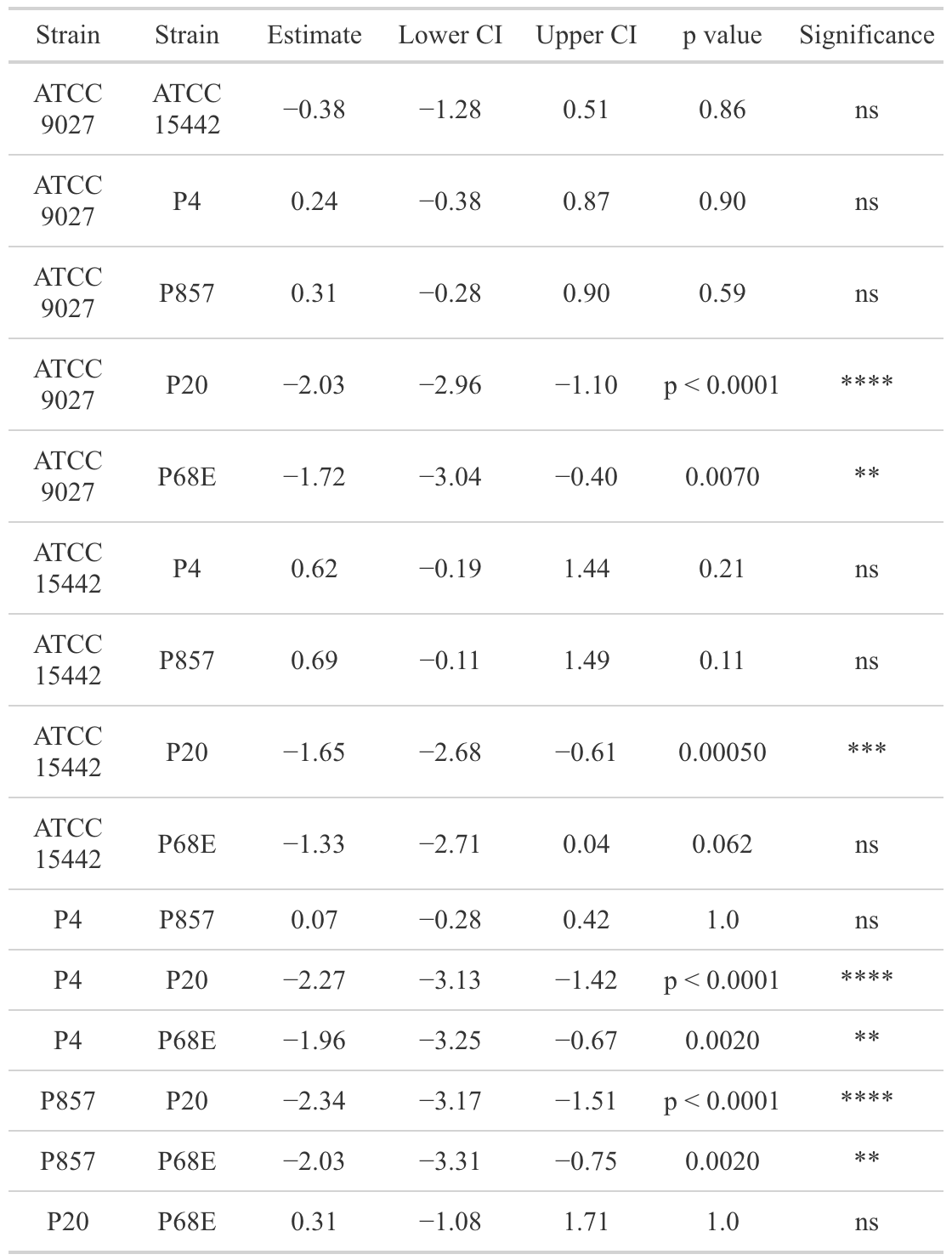


**Supplementary Table S3.** Parameters of the tested statistic of the differences in biofilm metabolic activity between particular *P. aeruginosa* strains (n=10). Welch’s ANOVA, followed by the Games-Howell test, was performed. Values of p<0.05 were considered significant, p<=0.05 was marked with one asterisk, p<=0.01 was marked with two asterisks, p<=0.001 was marked with three asterisks, and p<=0.0001 was marked with four asterisks. Ns- no significant differences, upper-lower Cl- confidence interval 95%.


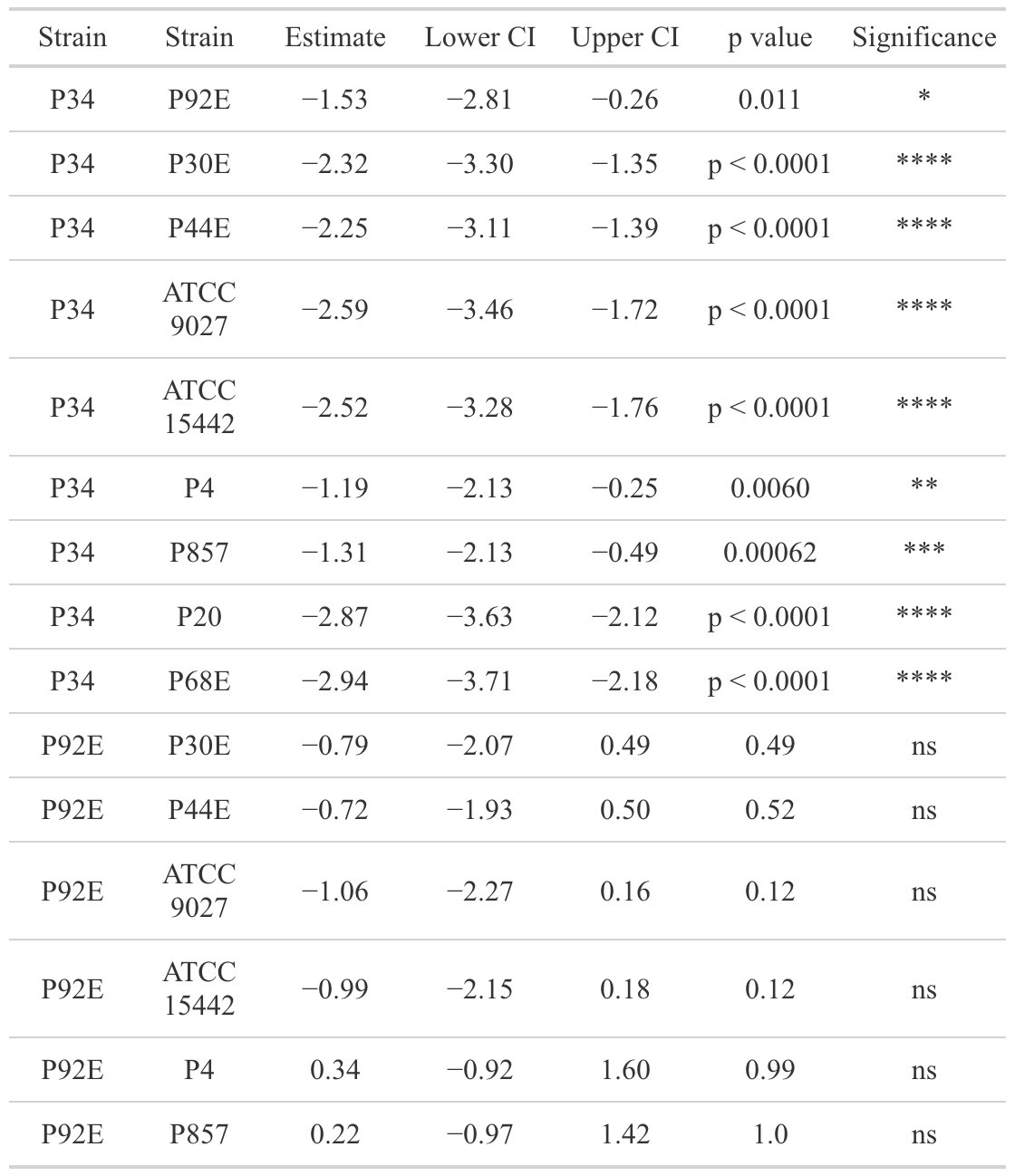


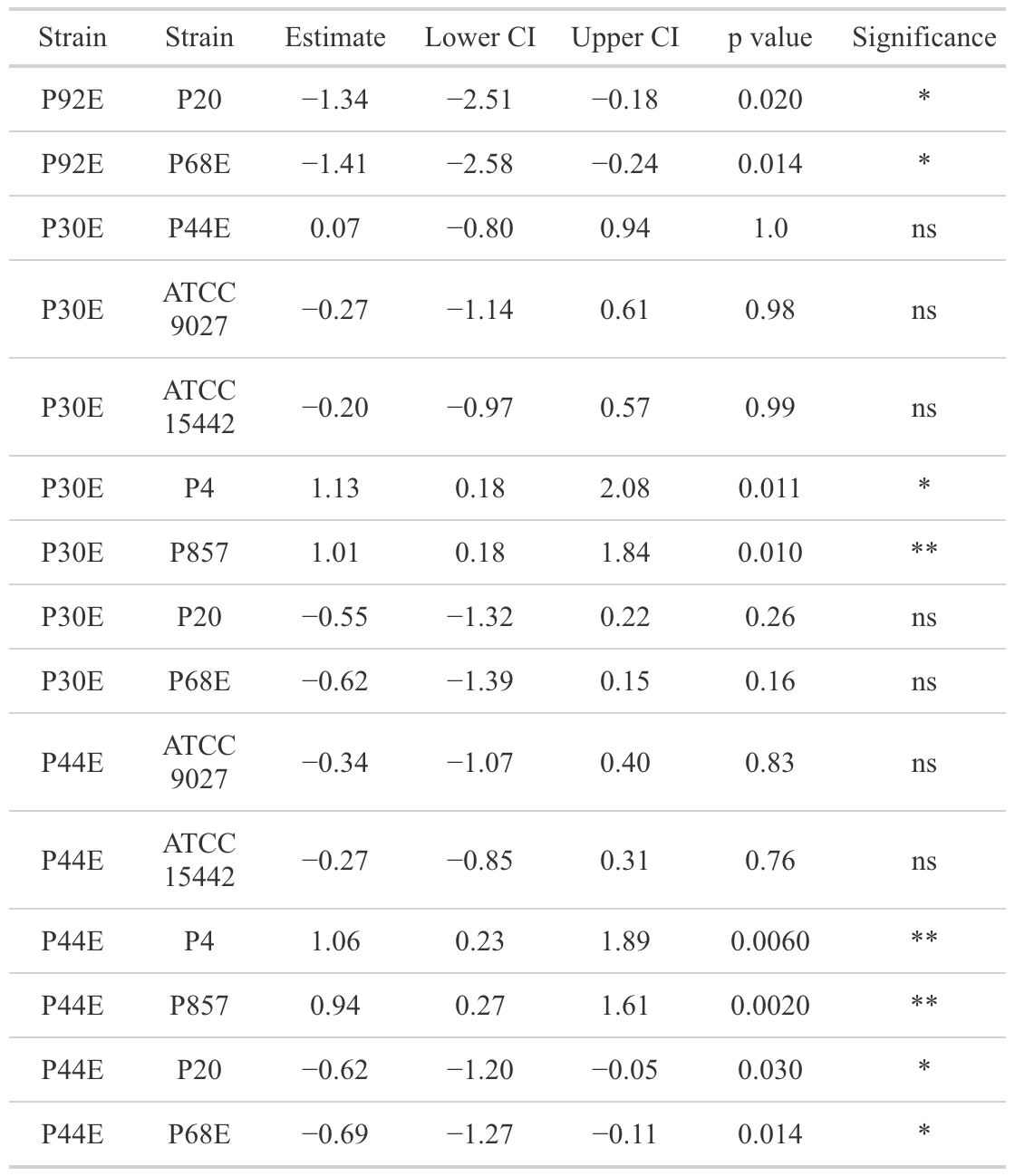


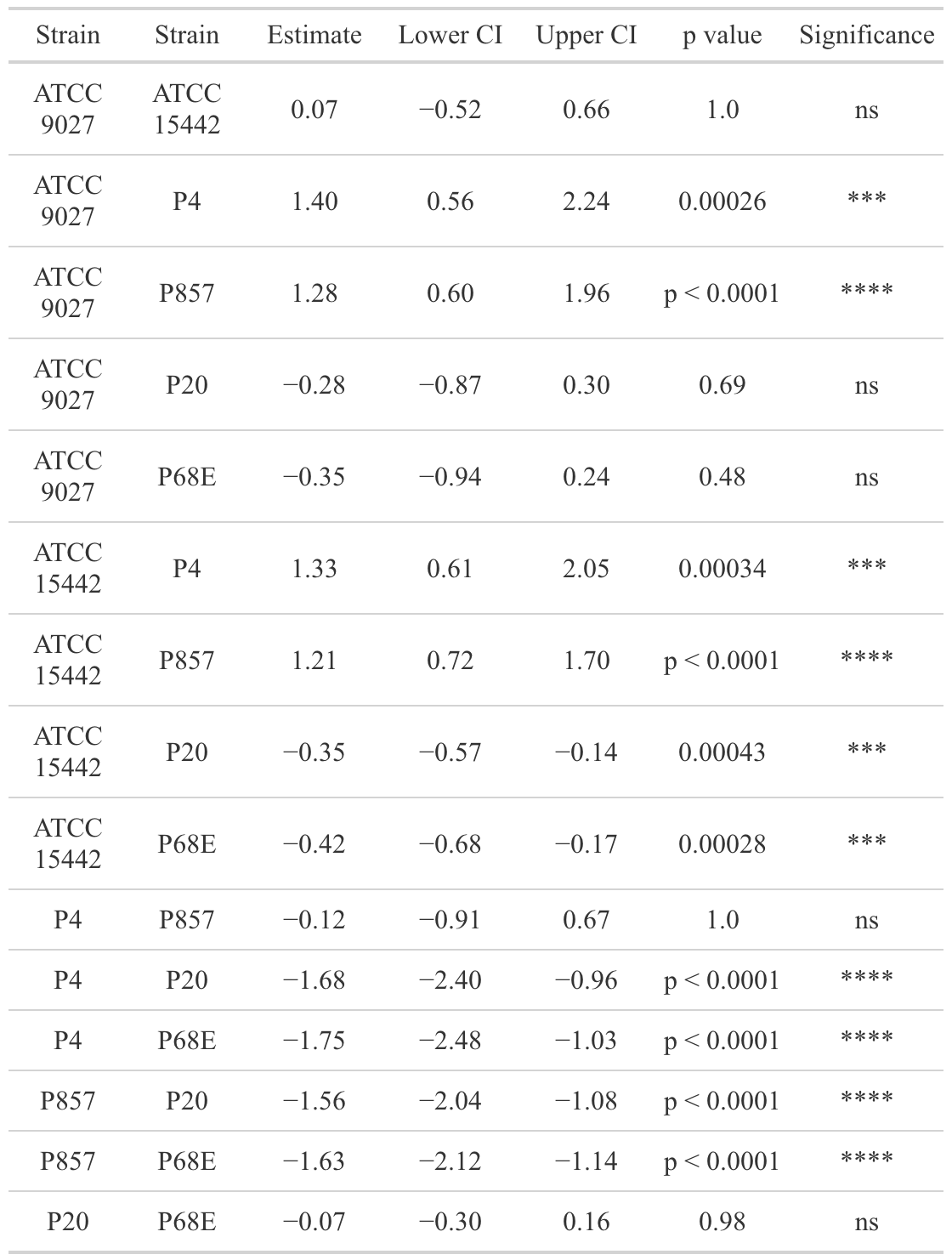


**Supplementary Table S4**. Parameters of the tested statistic of the differences in biofilm Colony-Forming Unit (CFU/mL) number between particular *P. aeruginosa* strains (n=10). Dunn’s test, followed by the Kruskal-Wallis test, was performed. Values of p<0.05 were considered significant, p<=0.05 was marked with one asterisk, and p<=0.01 was marked with two asterisks. Ns- no significant differences, N-data points.


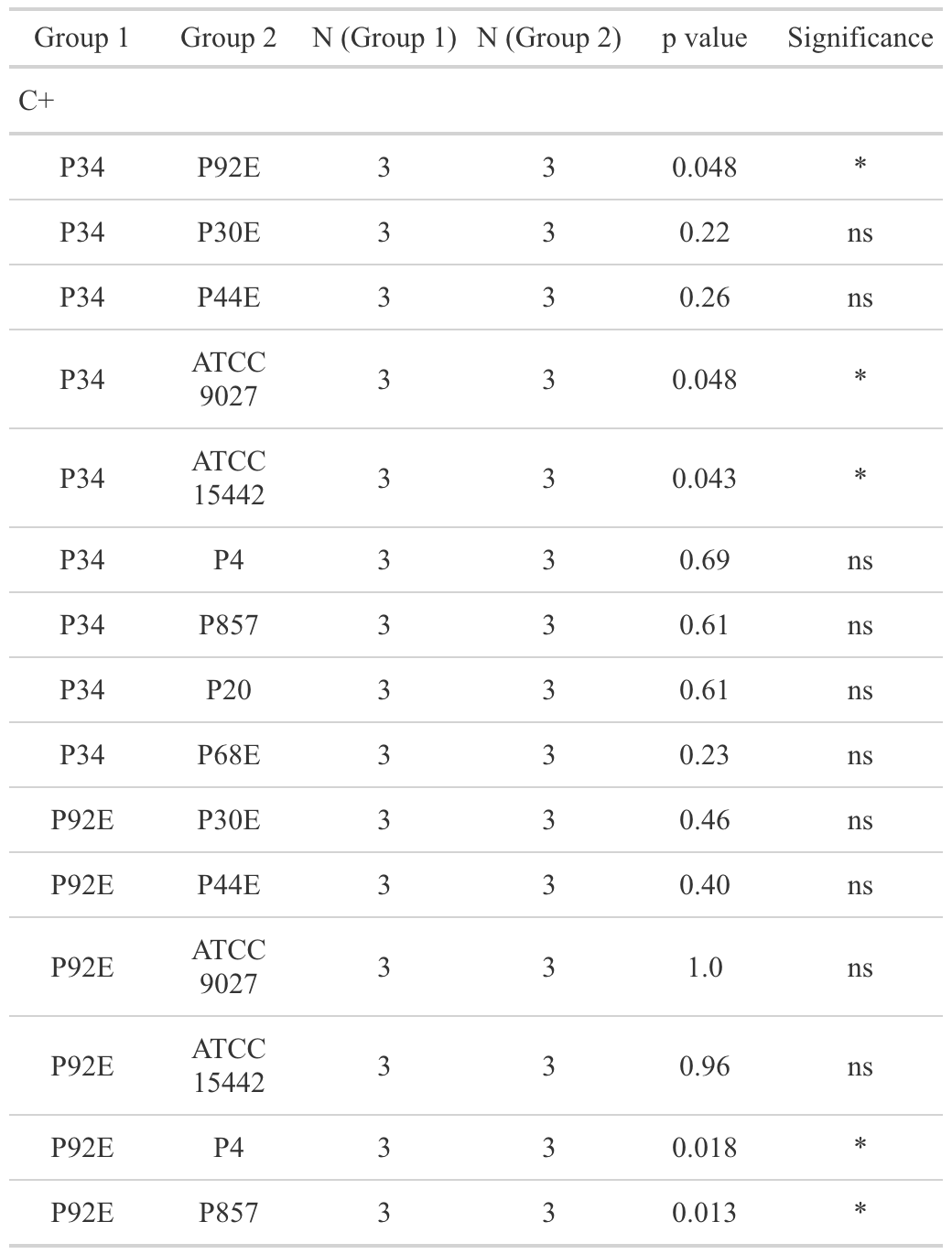


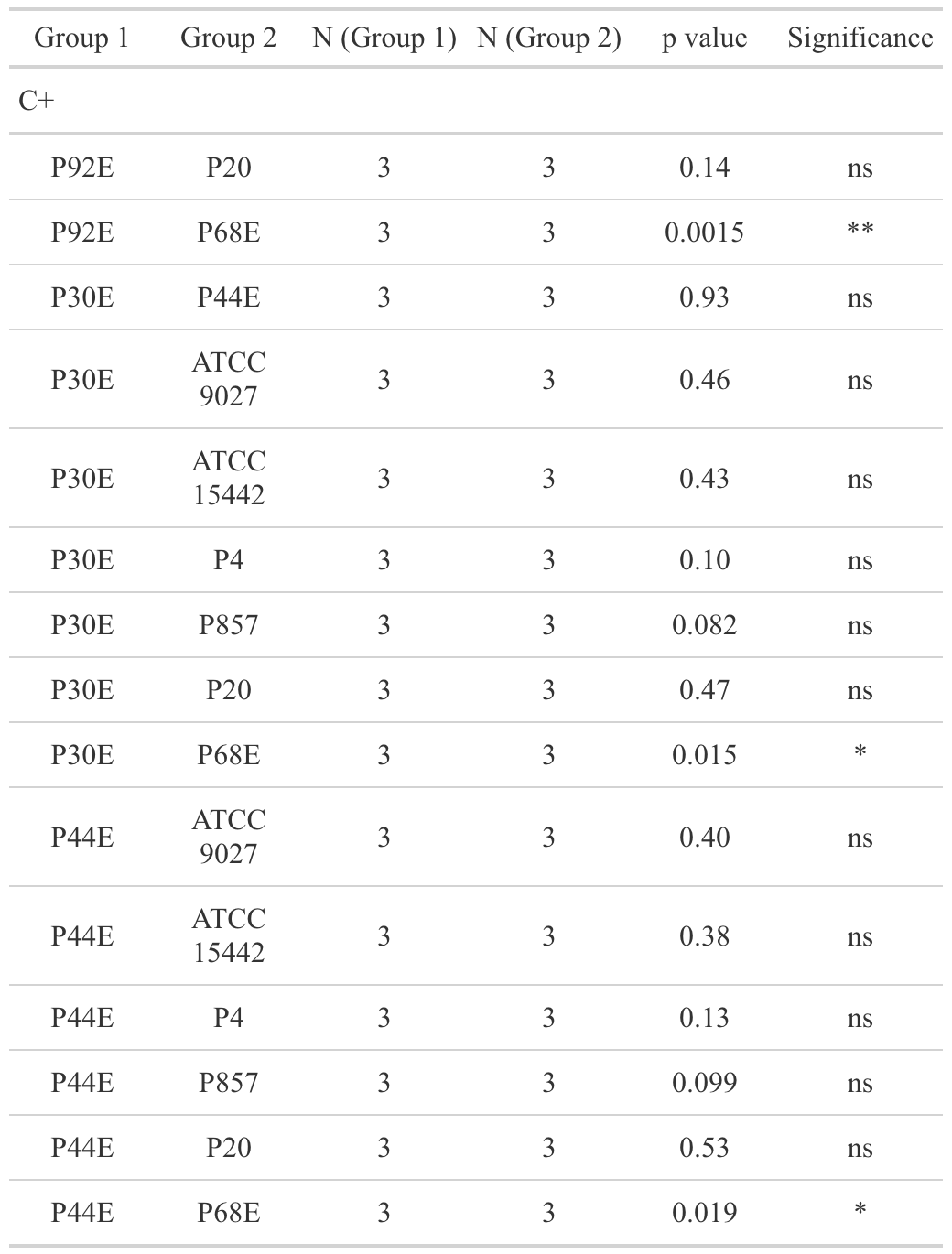


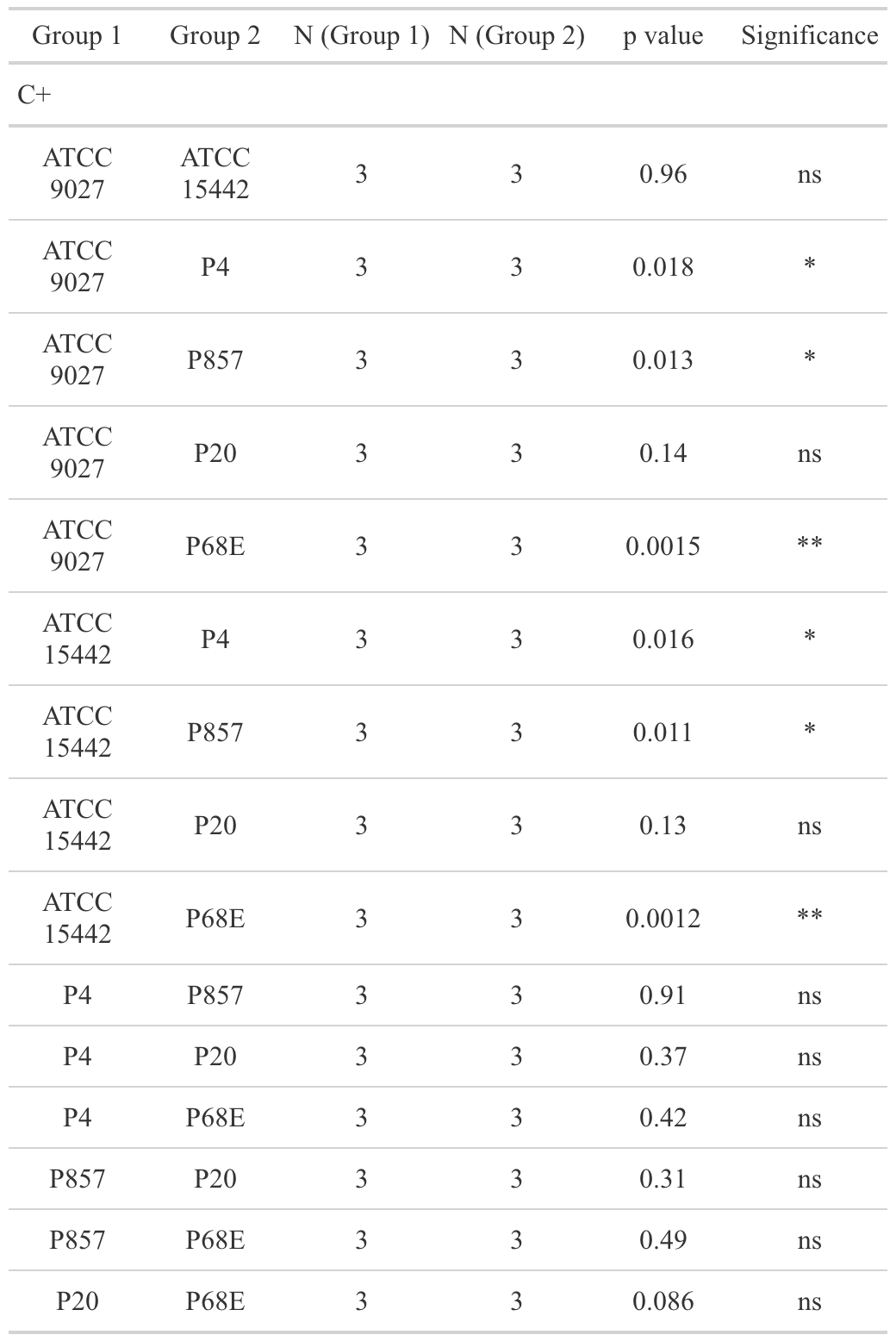


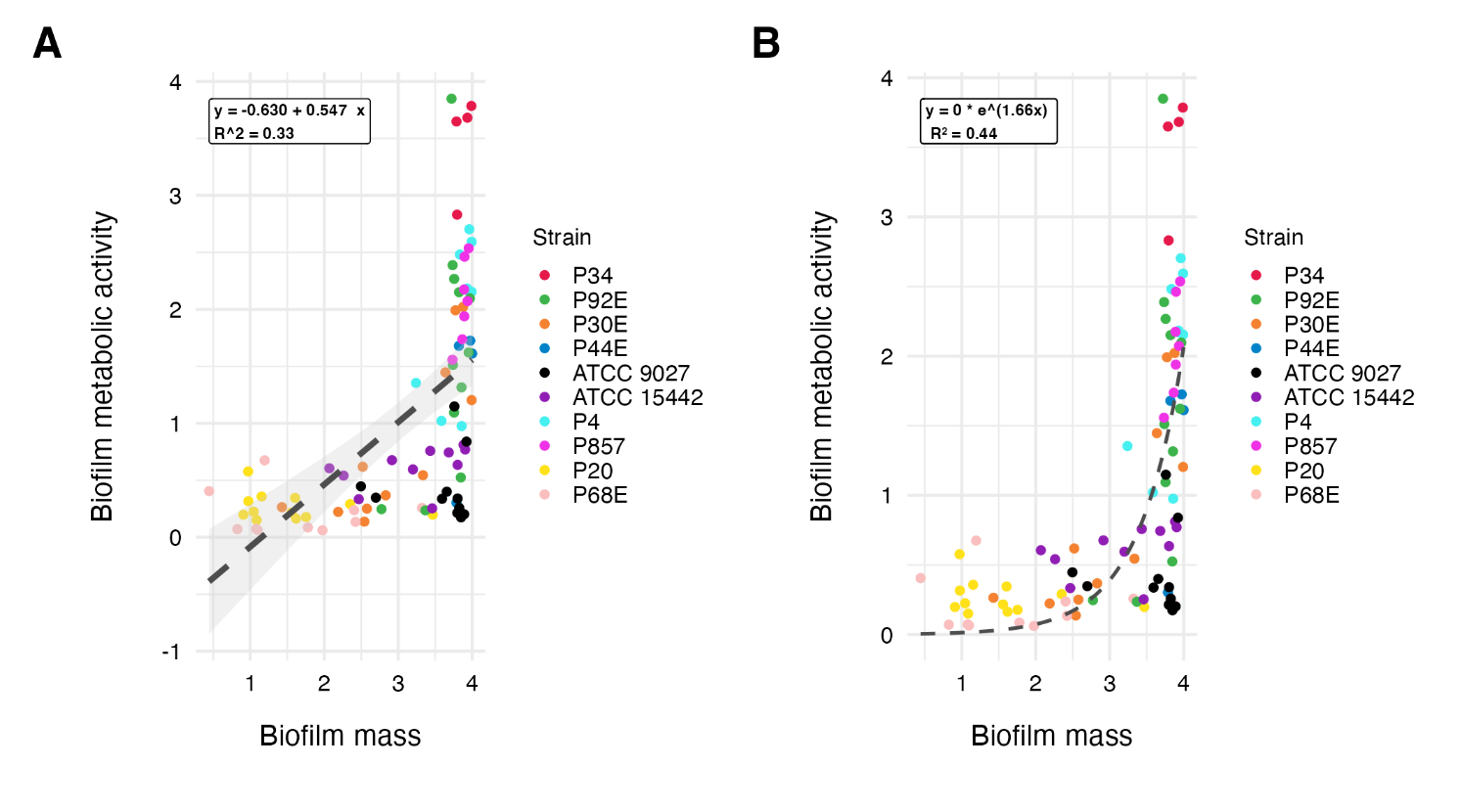
**Supplementary Figure S1.** Scatter plots of correlations of *P. aeruginosa* strains’ (n=10) biofilm mass and metabolic activity. Data fitted on linear trend line **(A)**. Data fitted on exponential trend line **(B)**. The equation for the line of best fit, and R^2^- coefficient of determination are present on the top left corners of the respective panels.

**Supplementary Table S5**. Parameters of the tested statistic of the differences in susceptibility to tested compounds between particular *P. aeruginosa* strains (n=10) assessed using modified disk diffusion method. Dunn’s test, followed by the Kruskal-Wallis test, was performed. Values of p<0.05 were considered significant, p<=0.05 was marked with one asterisk, and p<=0.01 was marked with two asterisks. Ns- no significant differences, N- data points, TEO- Thyme Essential Oil, PHMB- polyhexanide.


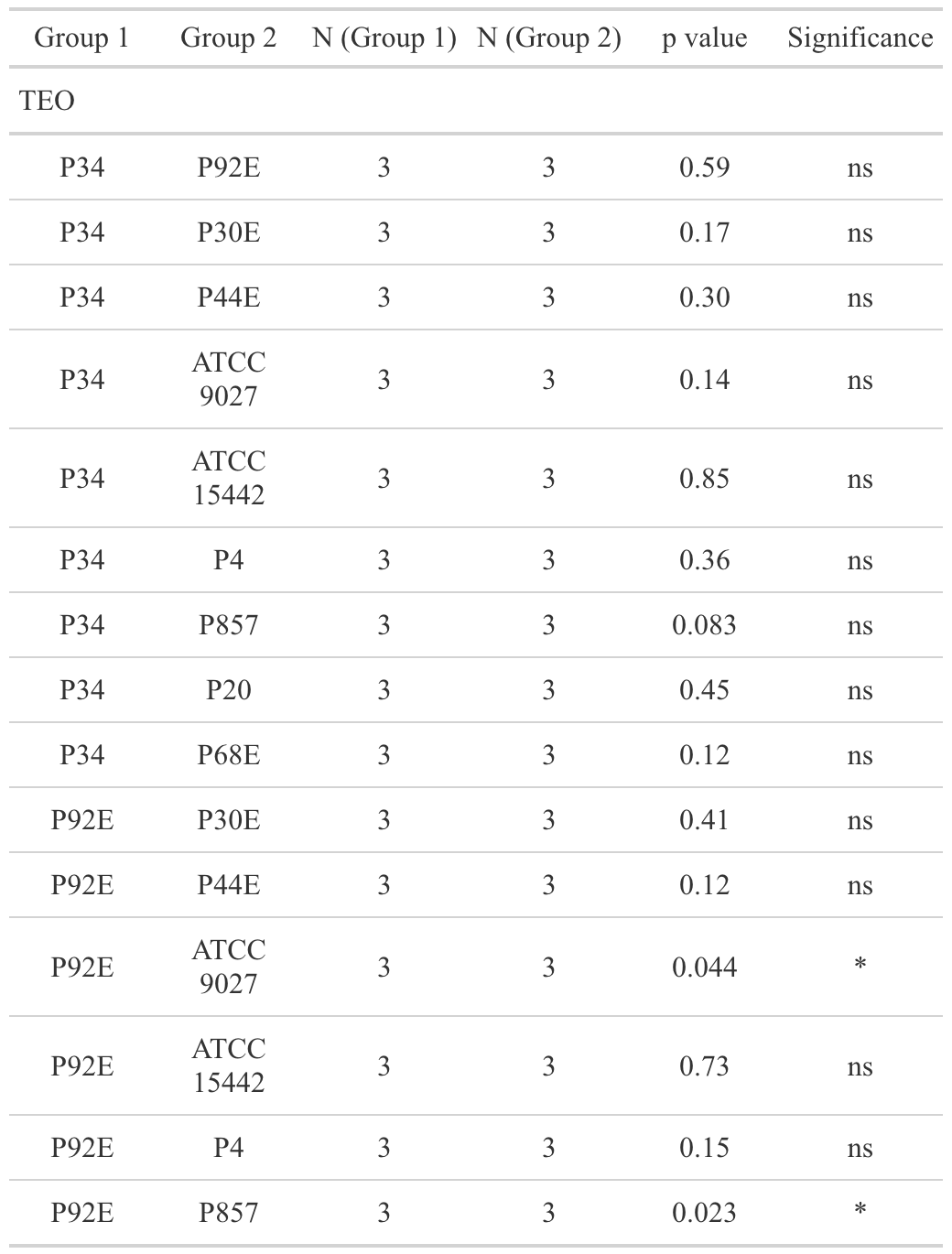


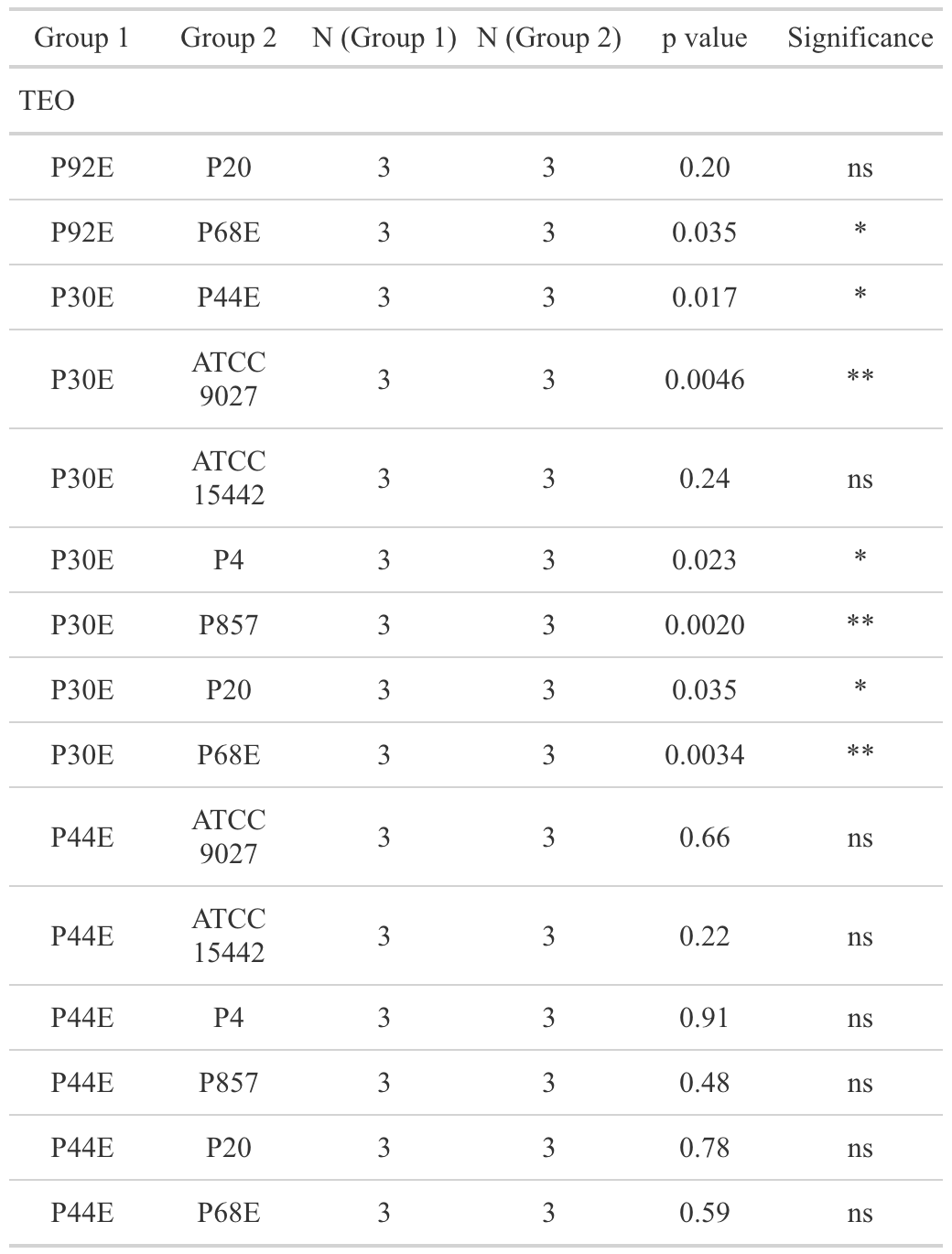


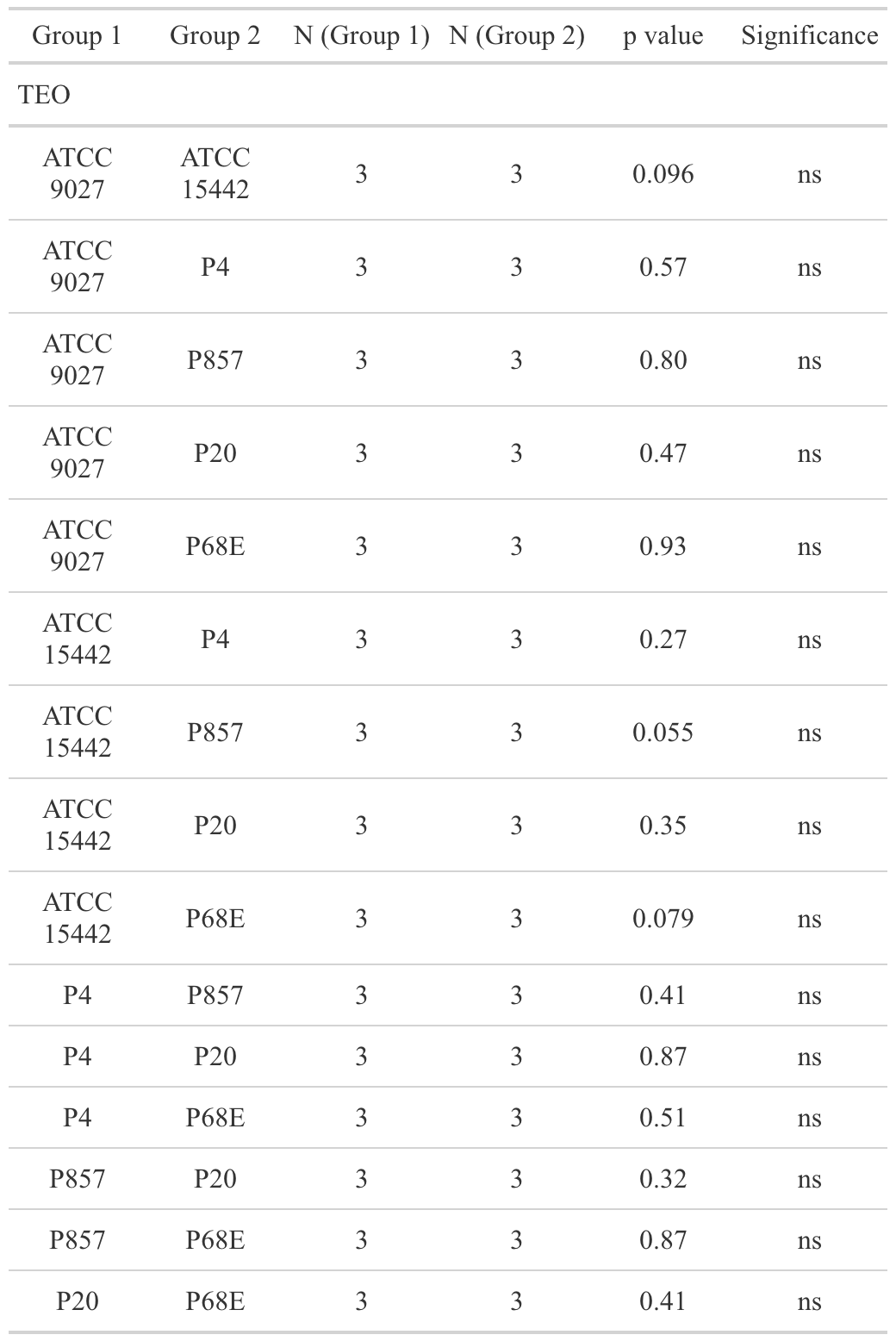


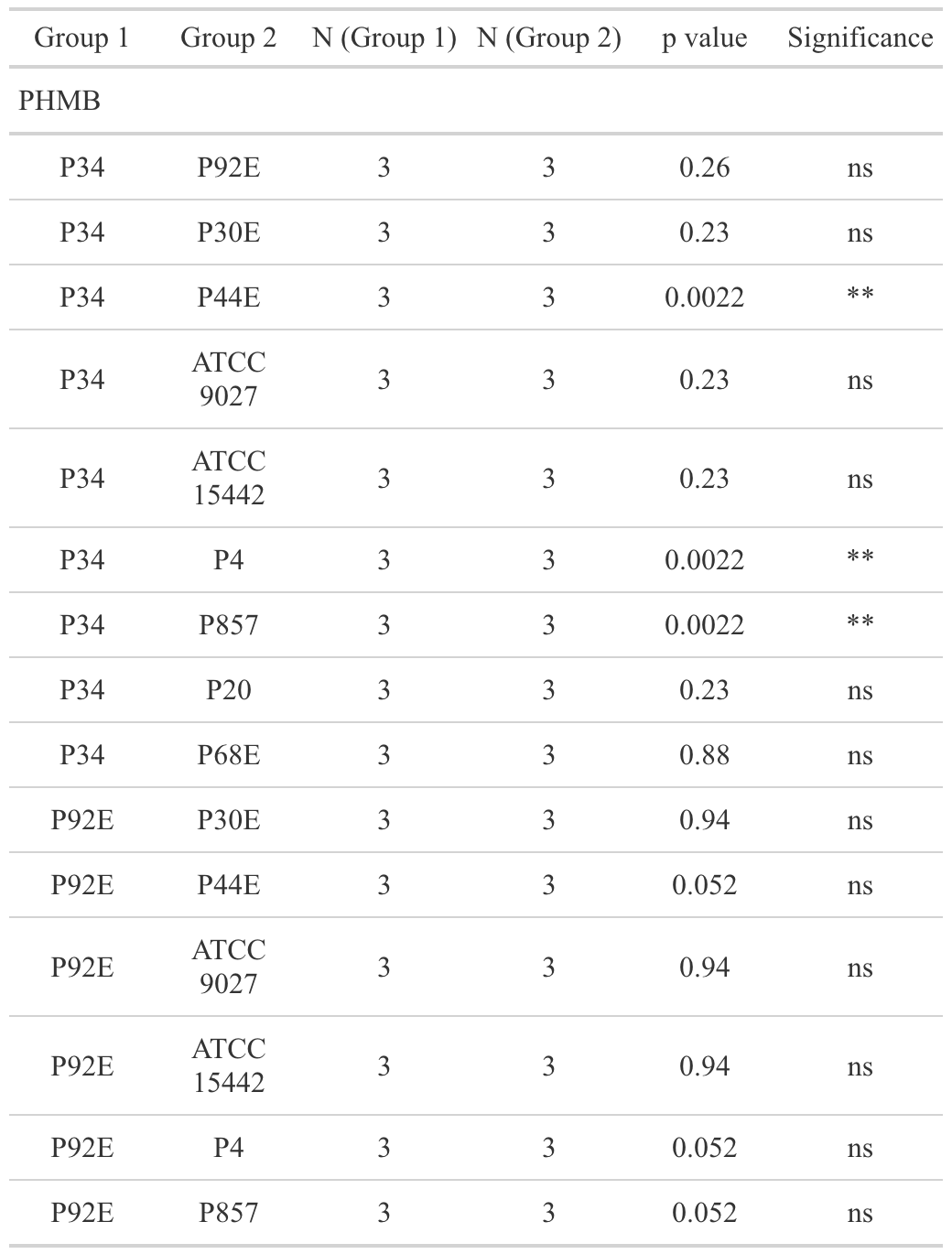


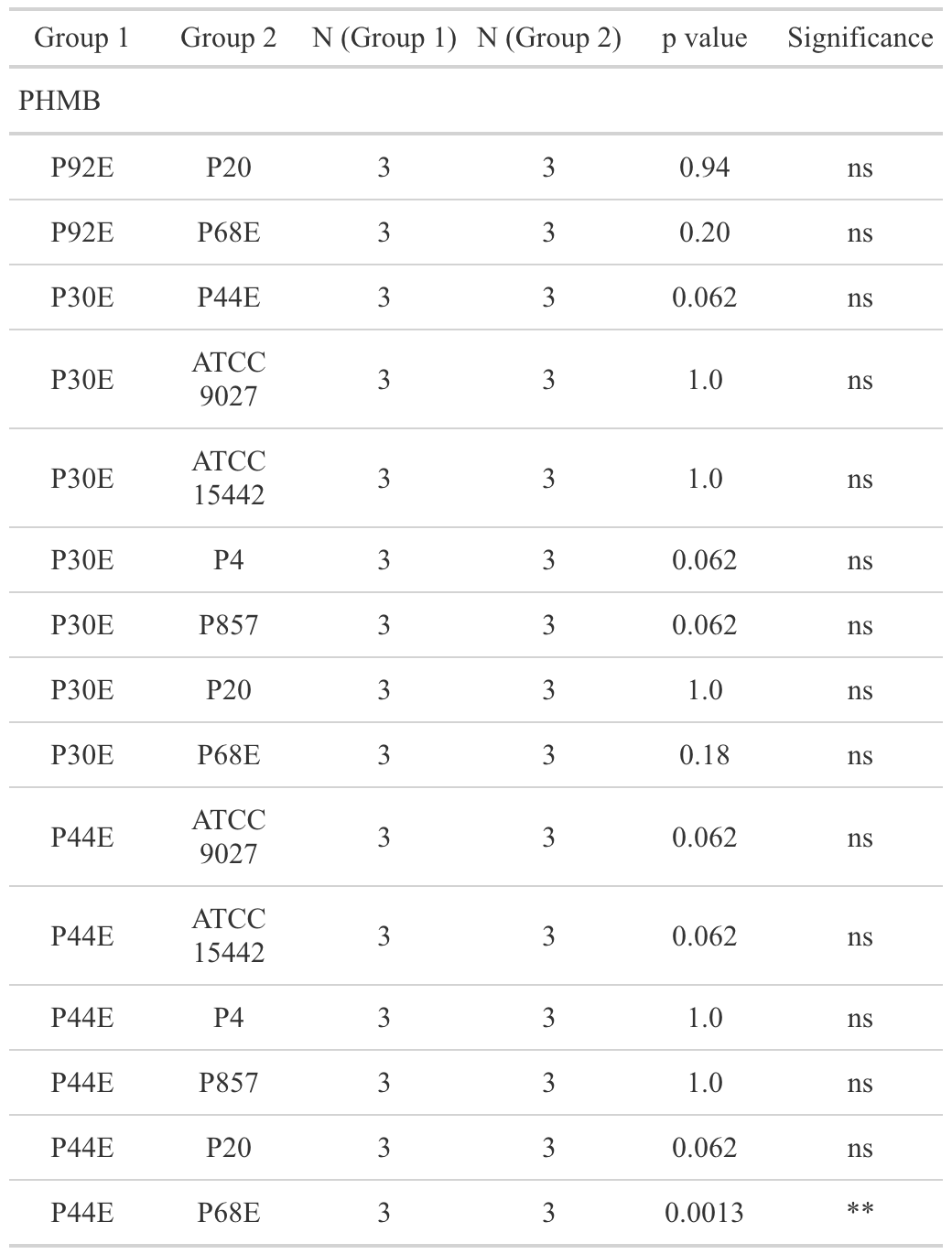


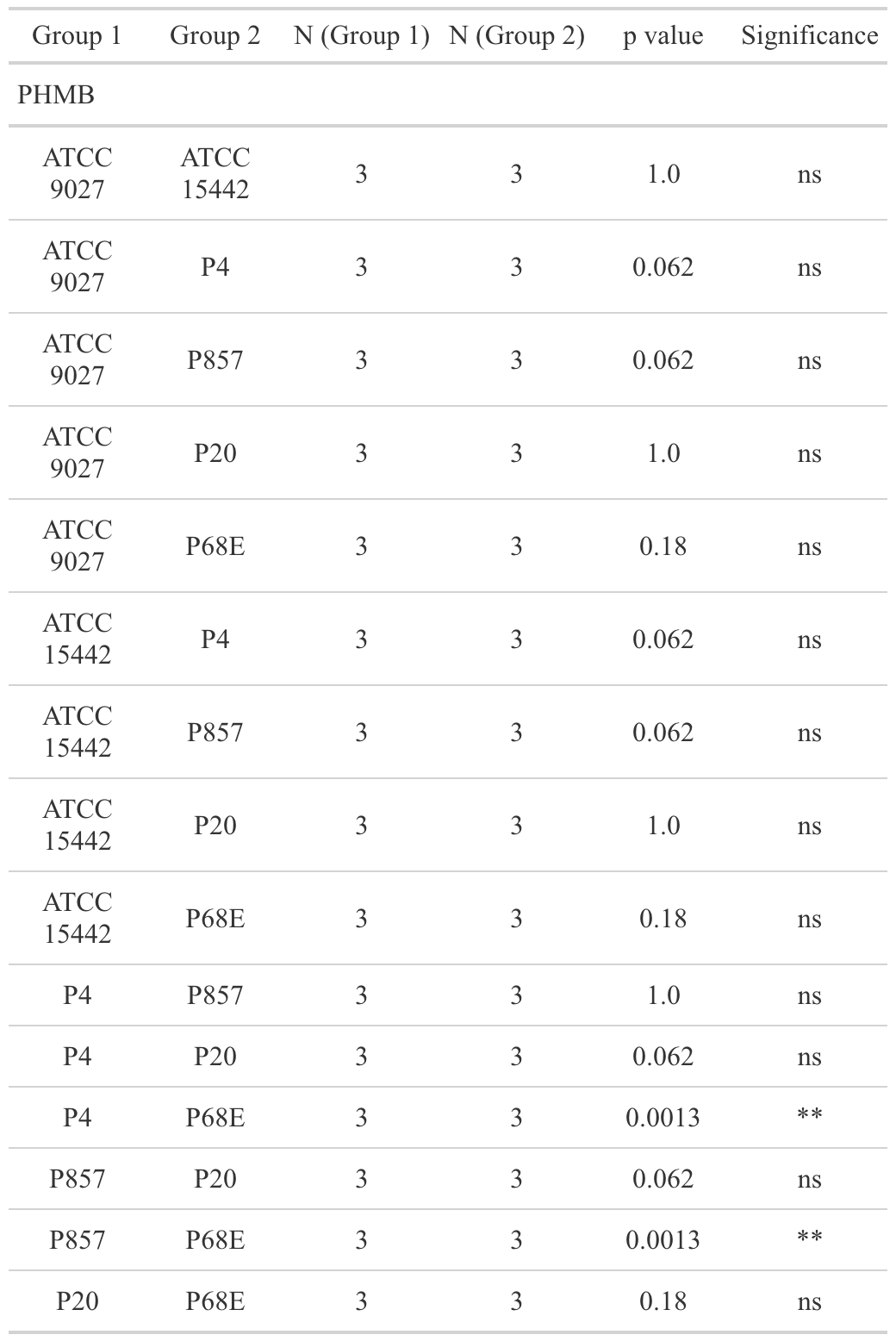


**Supplementary Table S6.** Parameters of the tested statistic of the differences in susceptibility to tested compounds between particular *P. aeruginosa* strains (n=10) assessed with a microdilution method. Dunn’s test, followed by the Kruskal-Wallis test, was performed. Values of p<0.05 were considered significant, p<=0.05 was marked with one asterisk, p<=0.01 was marked with two asterisks, and p<=0.001 was marked with three asterisks. Ns- no significant differences, N- data points, TEO- Thyme Essential Oil, PHMB- polyhexanide.


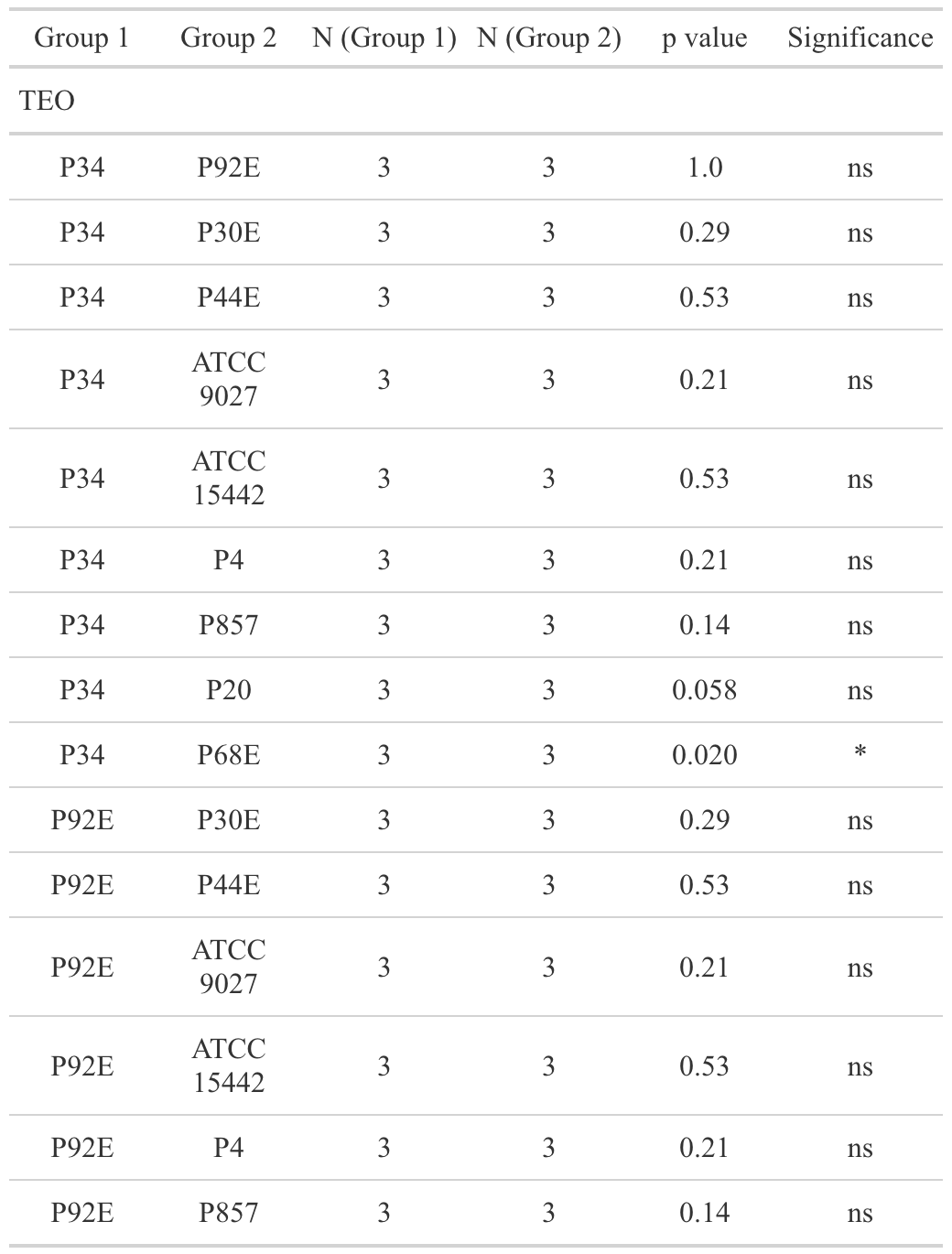


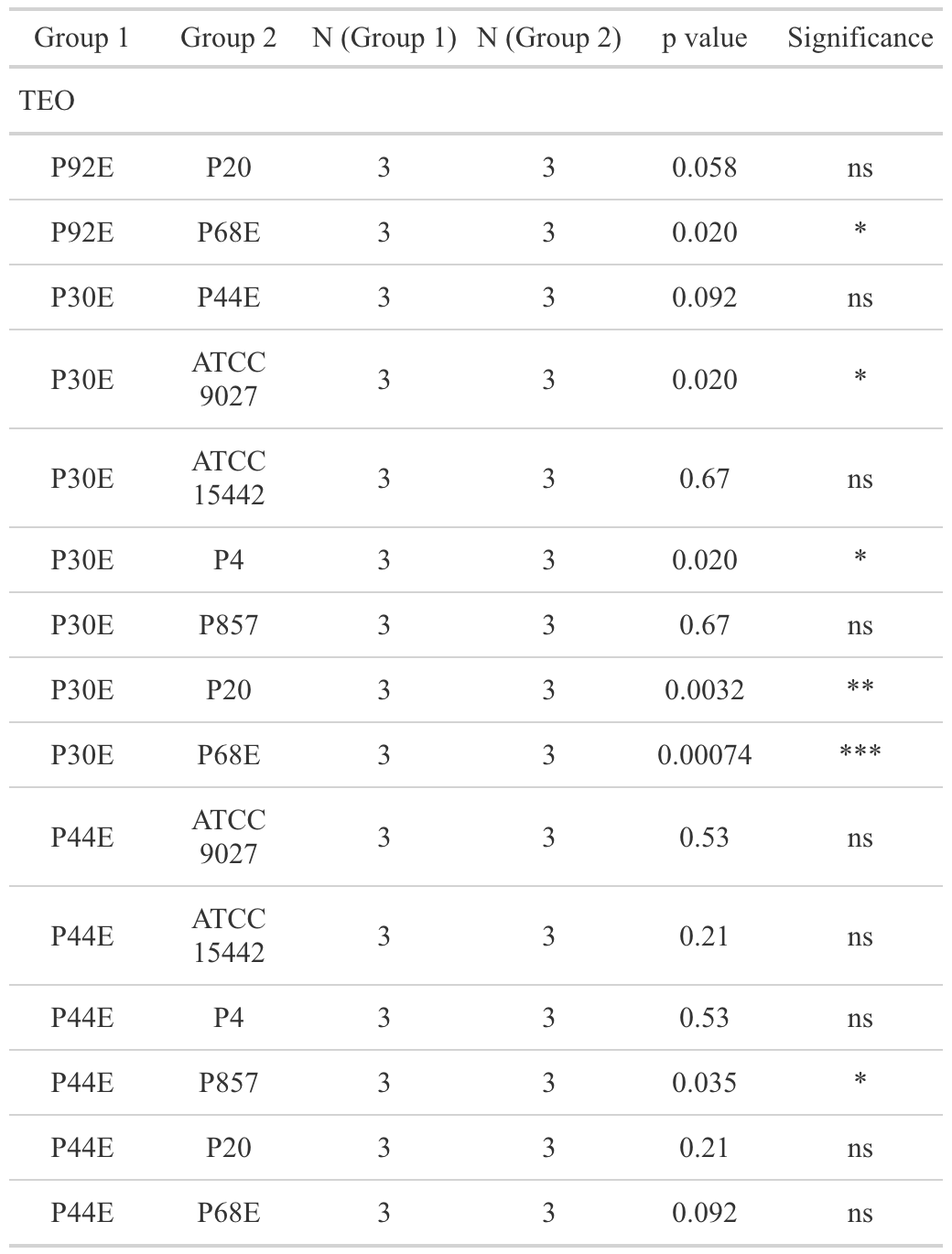


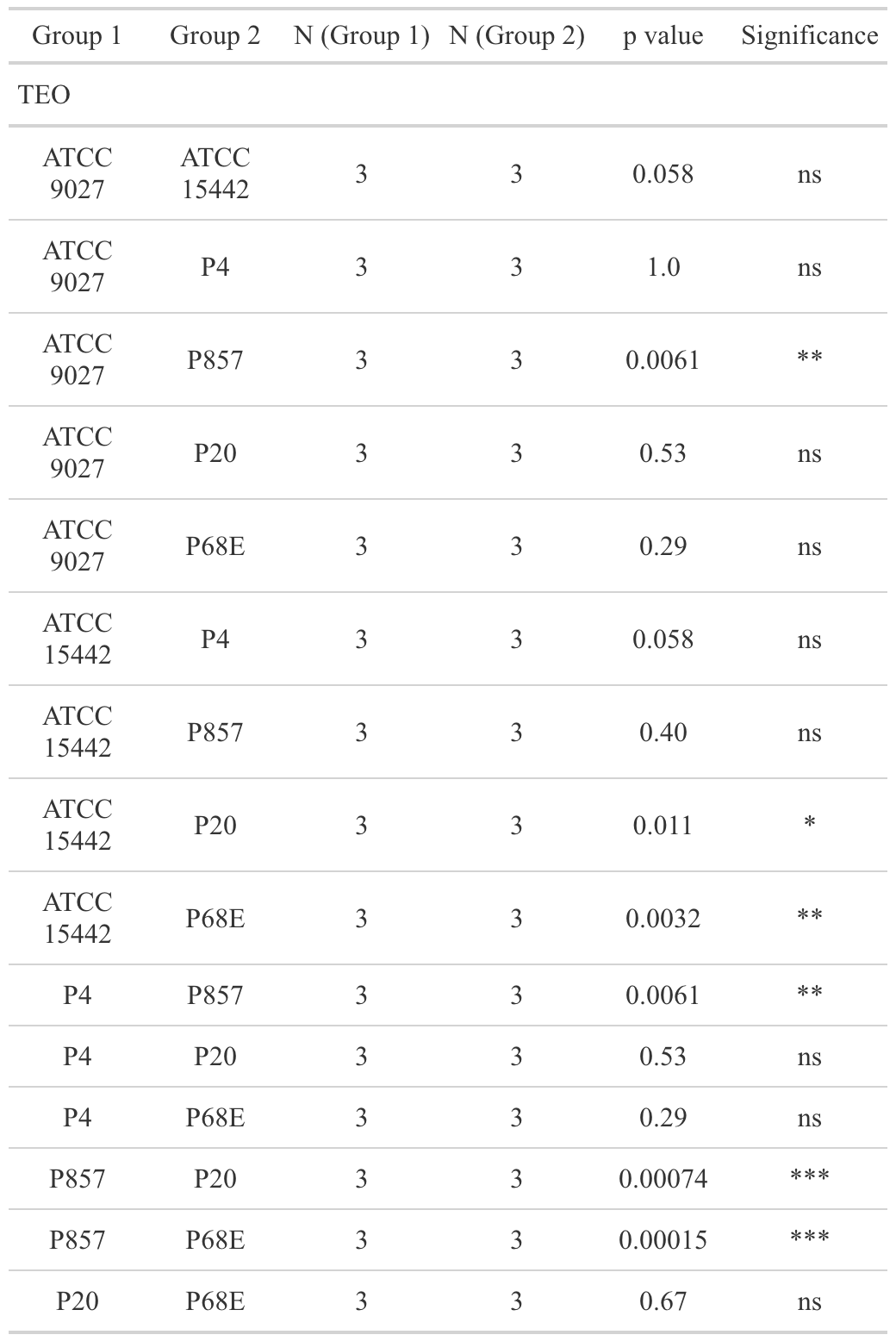


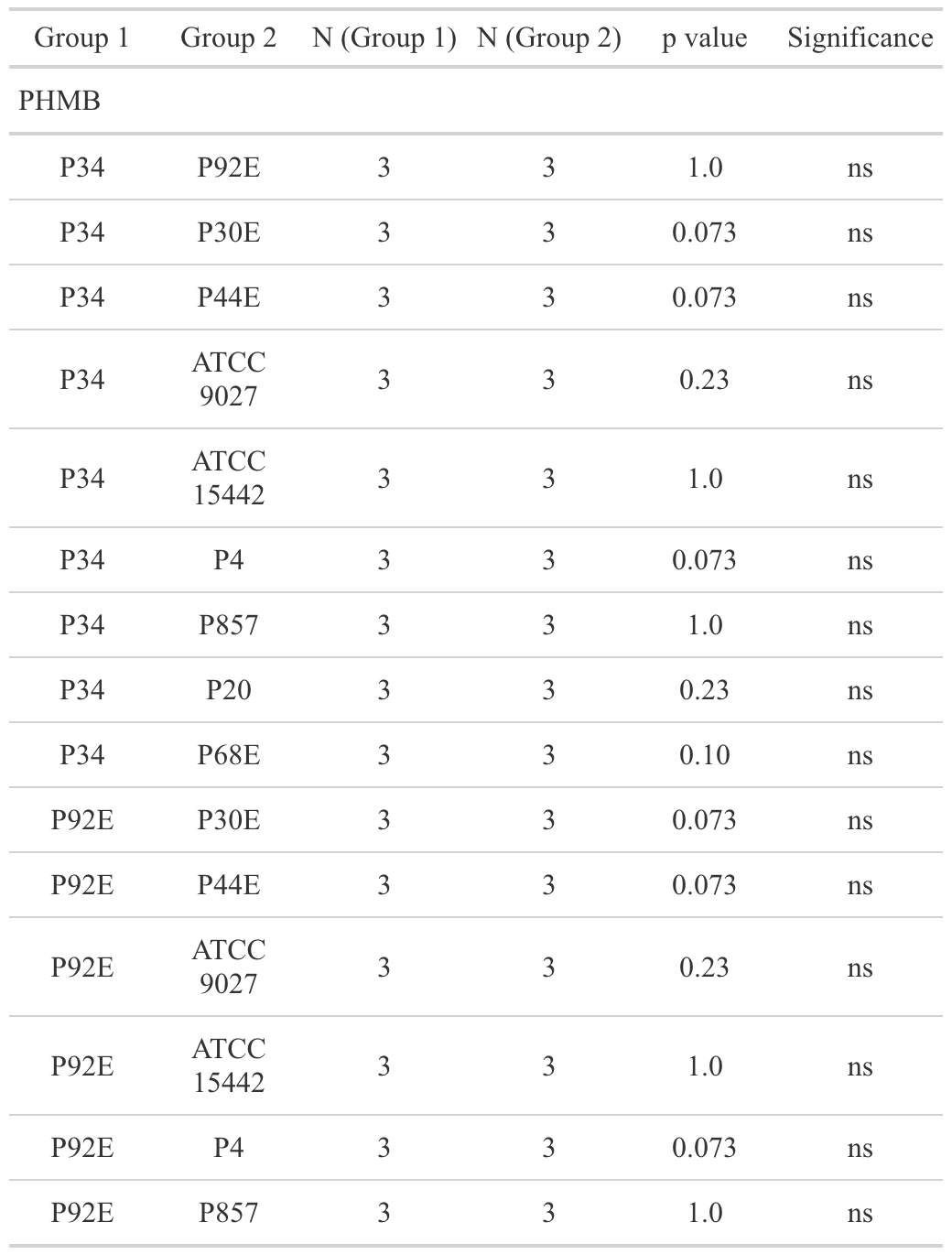


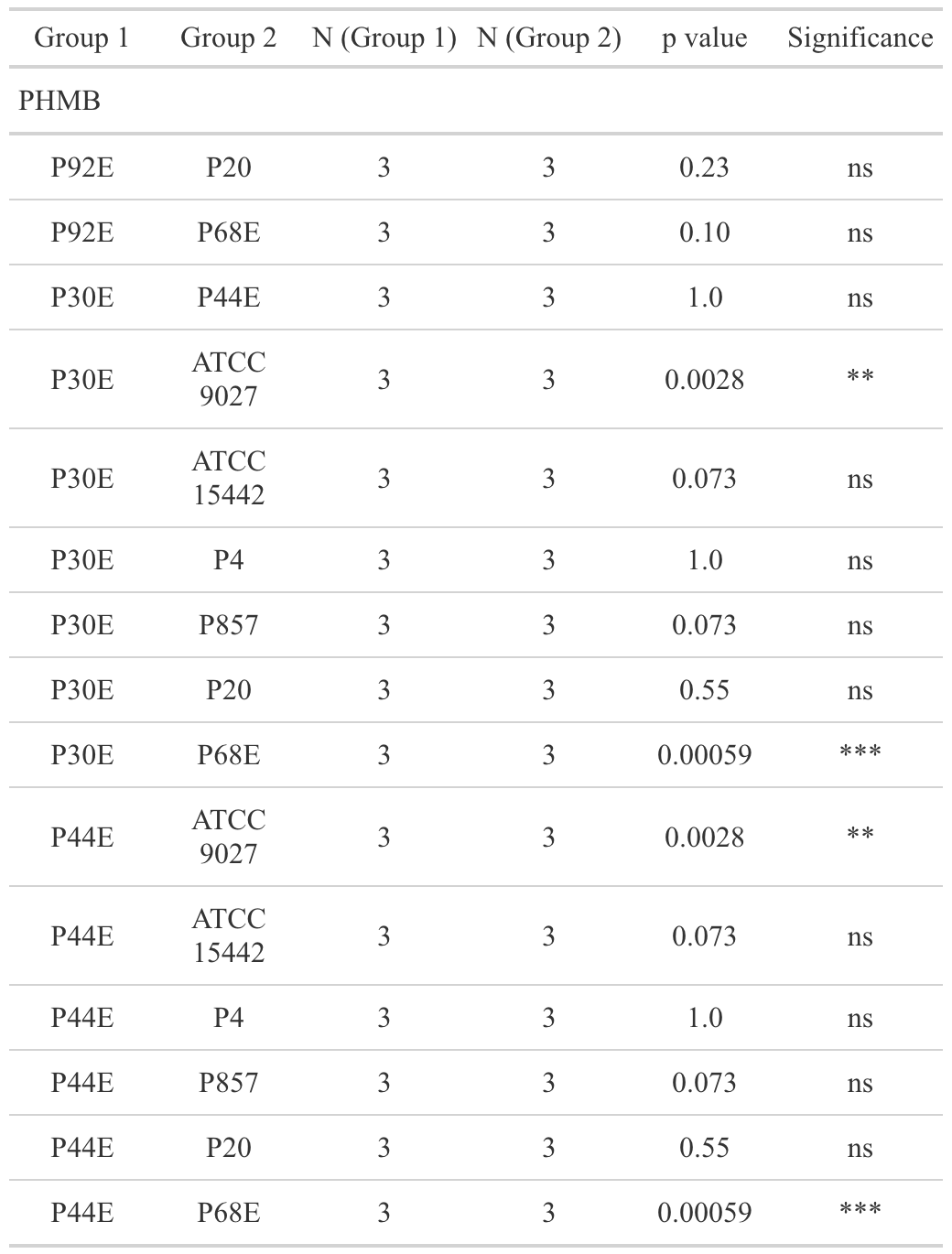


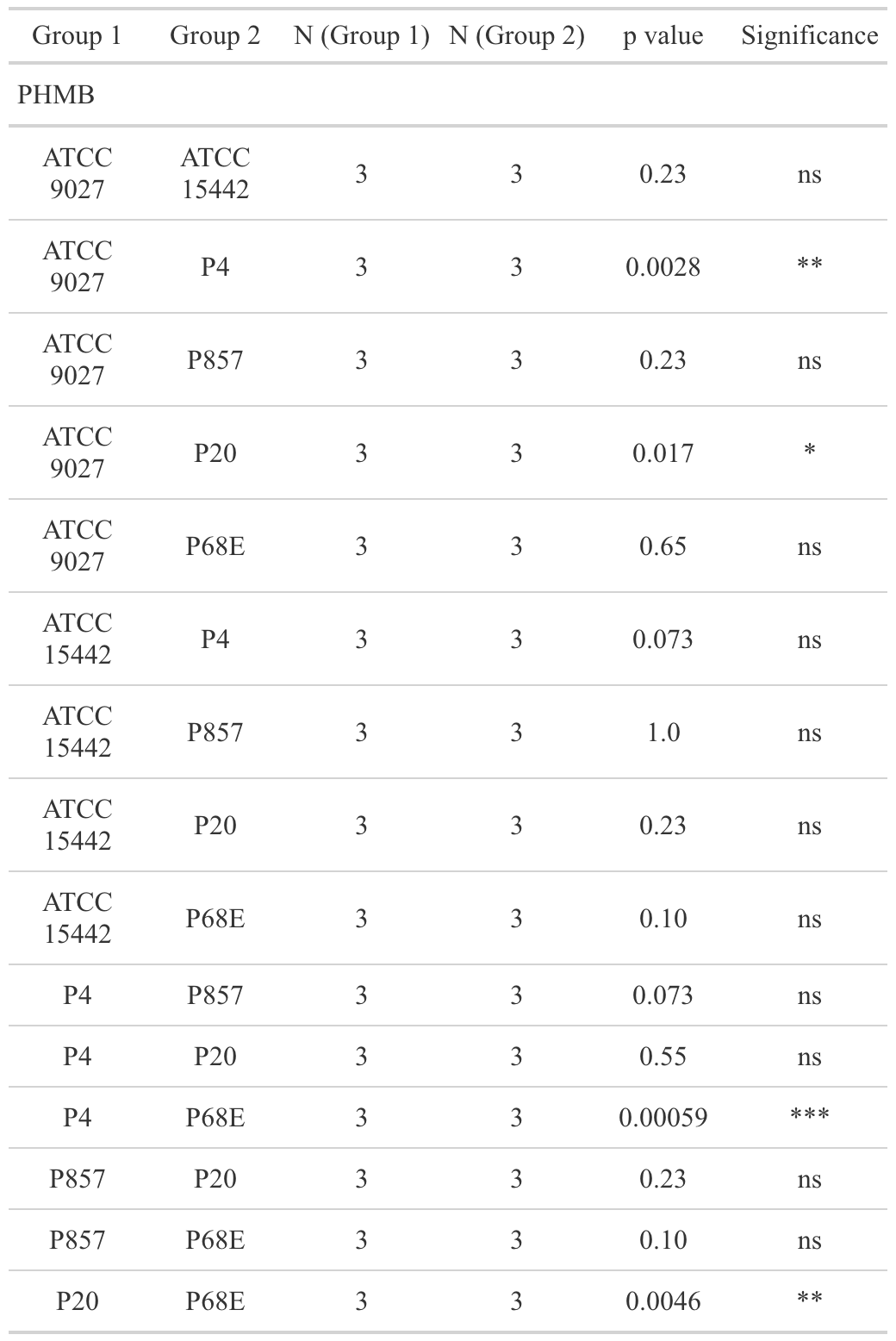


**Supplementary Table S7.** Biofilm CFU/mL values reduction across *P. aeruginosa* (n=10) strains and conditions. TEO- Thyme Essential Oil, PHMB- polyhexanide, CFU- Colony-Forming Unit.


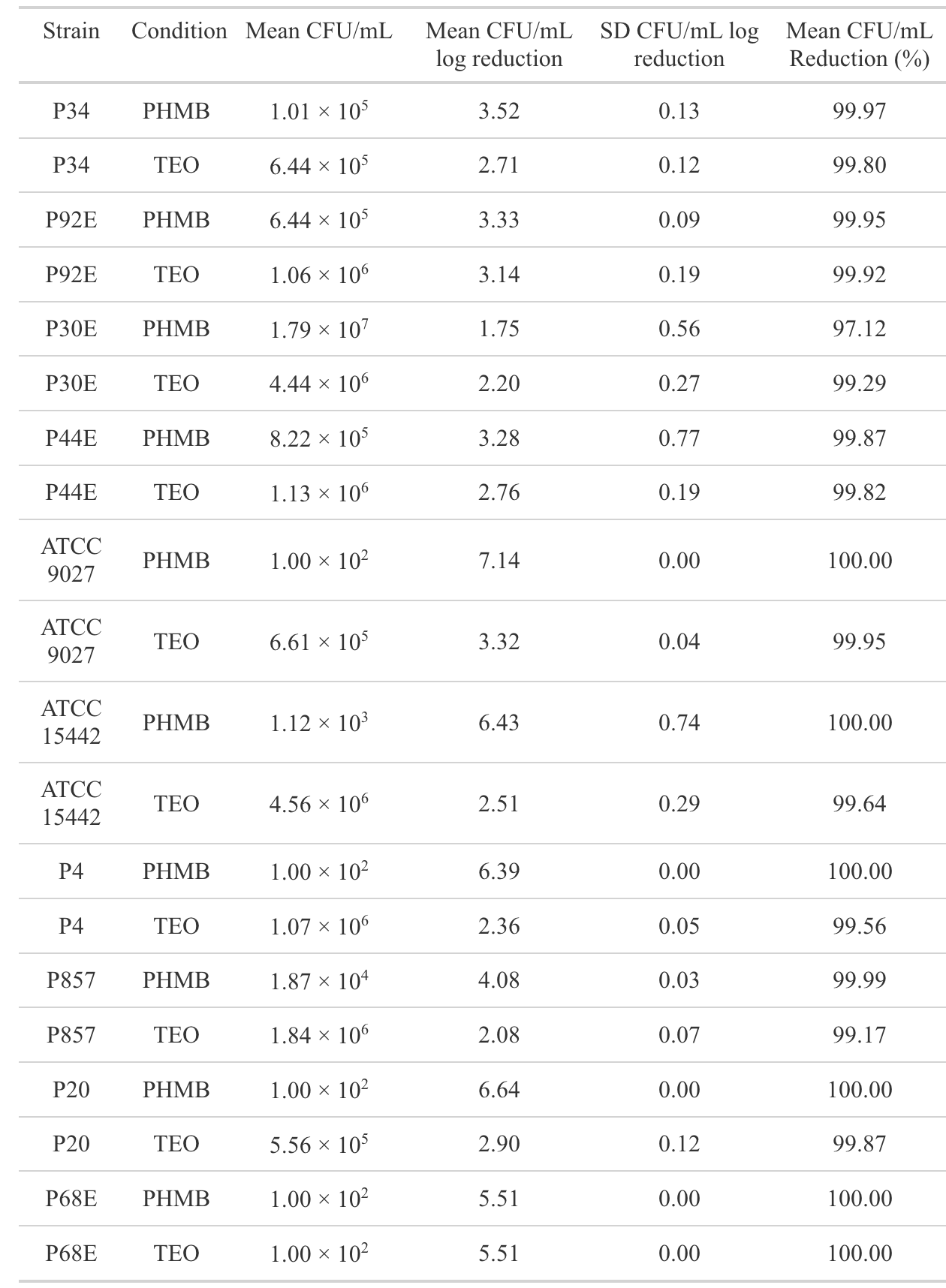


**Supplementary Table S8.** Parameters of the tested statistic of the differences in susceptibility to tested compounds between particular *P. aeruginosa* (n=10) strains expressed as biofilm reduction (%). Dunn’s test, followed by the Kruskal-Wallis test, was performed. Values of p<0.05 were considered significant, p<=0.05 was marked with one asterisk, p<=0.01 was marked with two asterisks, and p<=0.001 was marked with three asterisks. Ns- no significant differences, N- data points, TEO- Thyme Essential Oil, PHMB- polyhexanide.


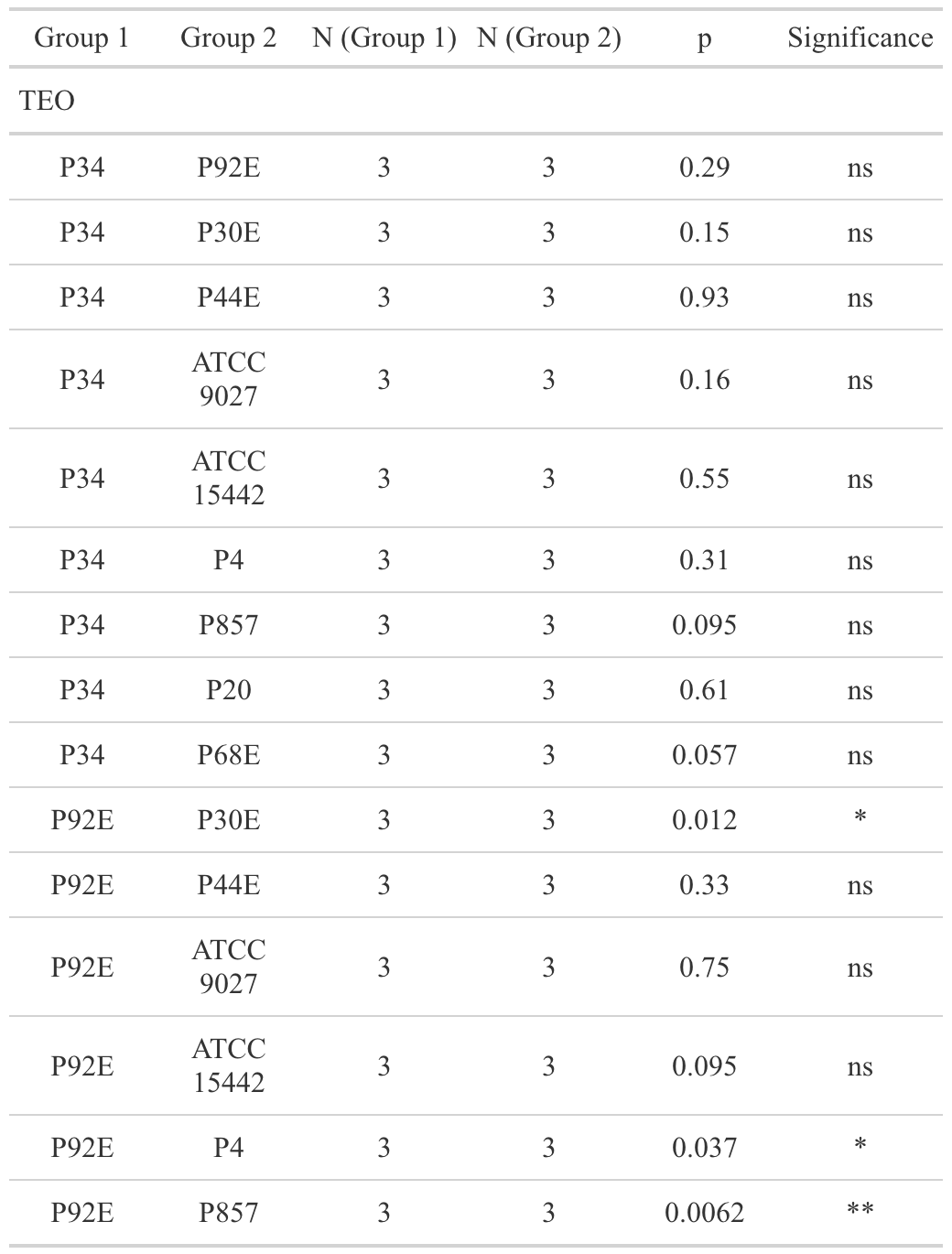


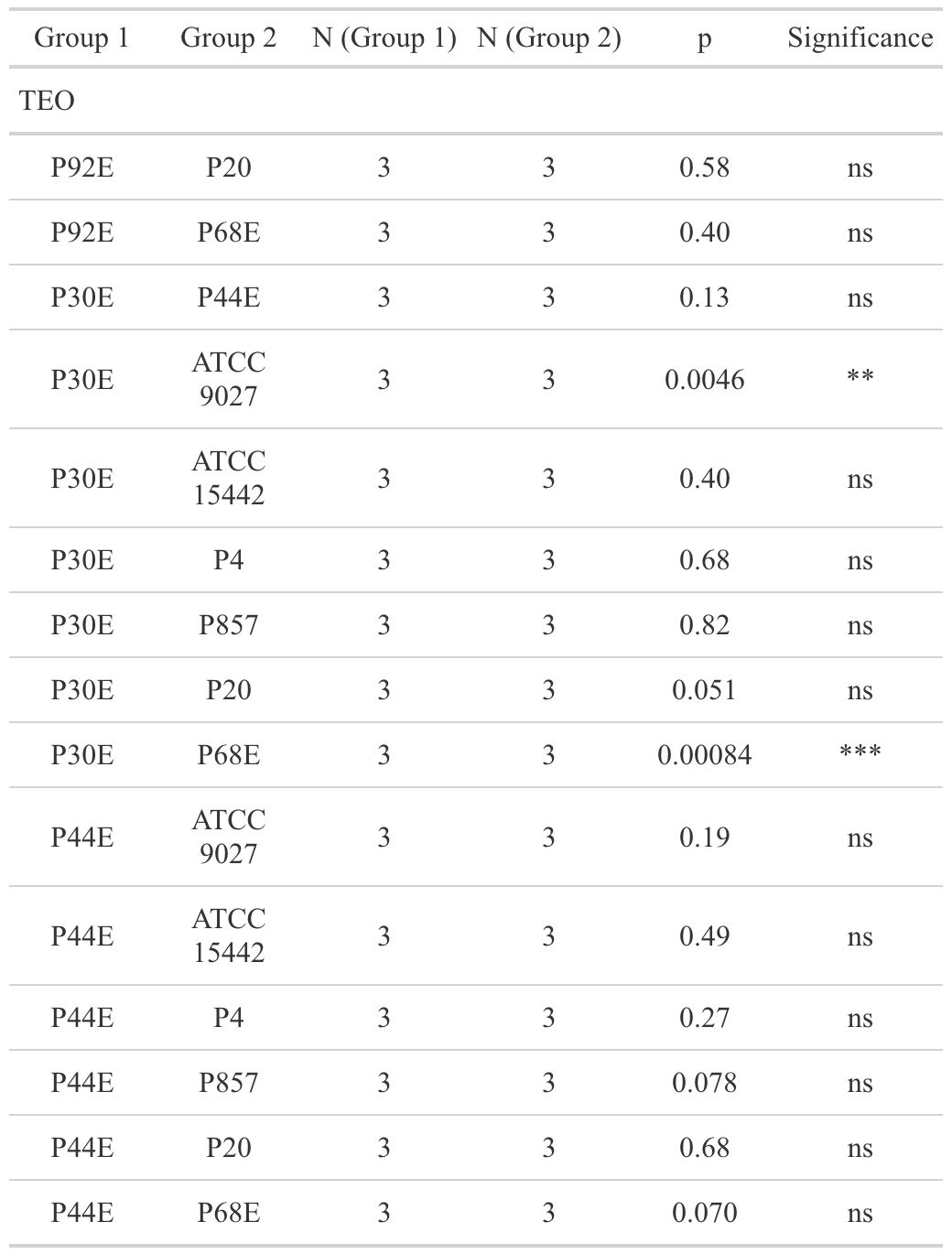


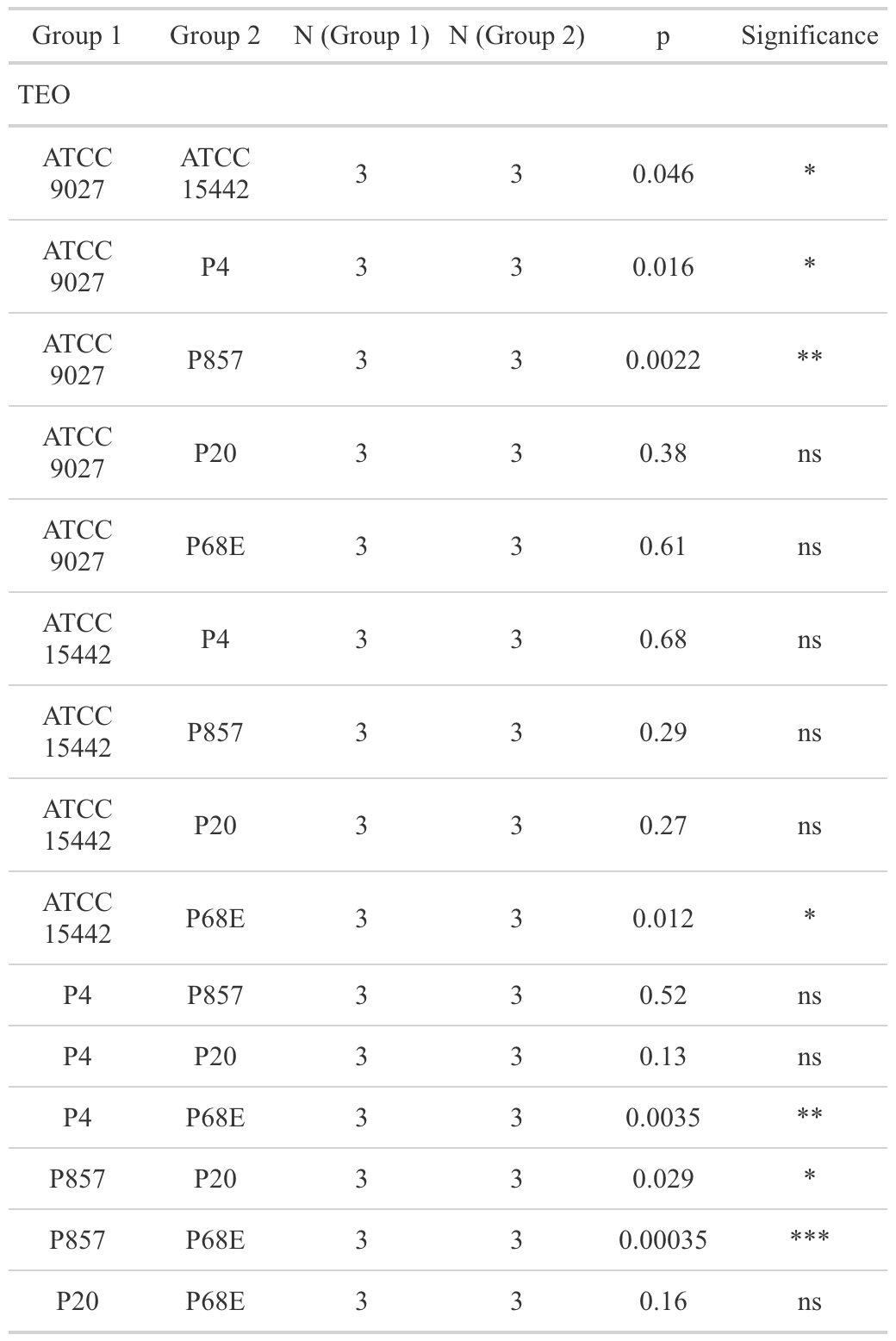


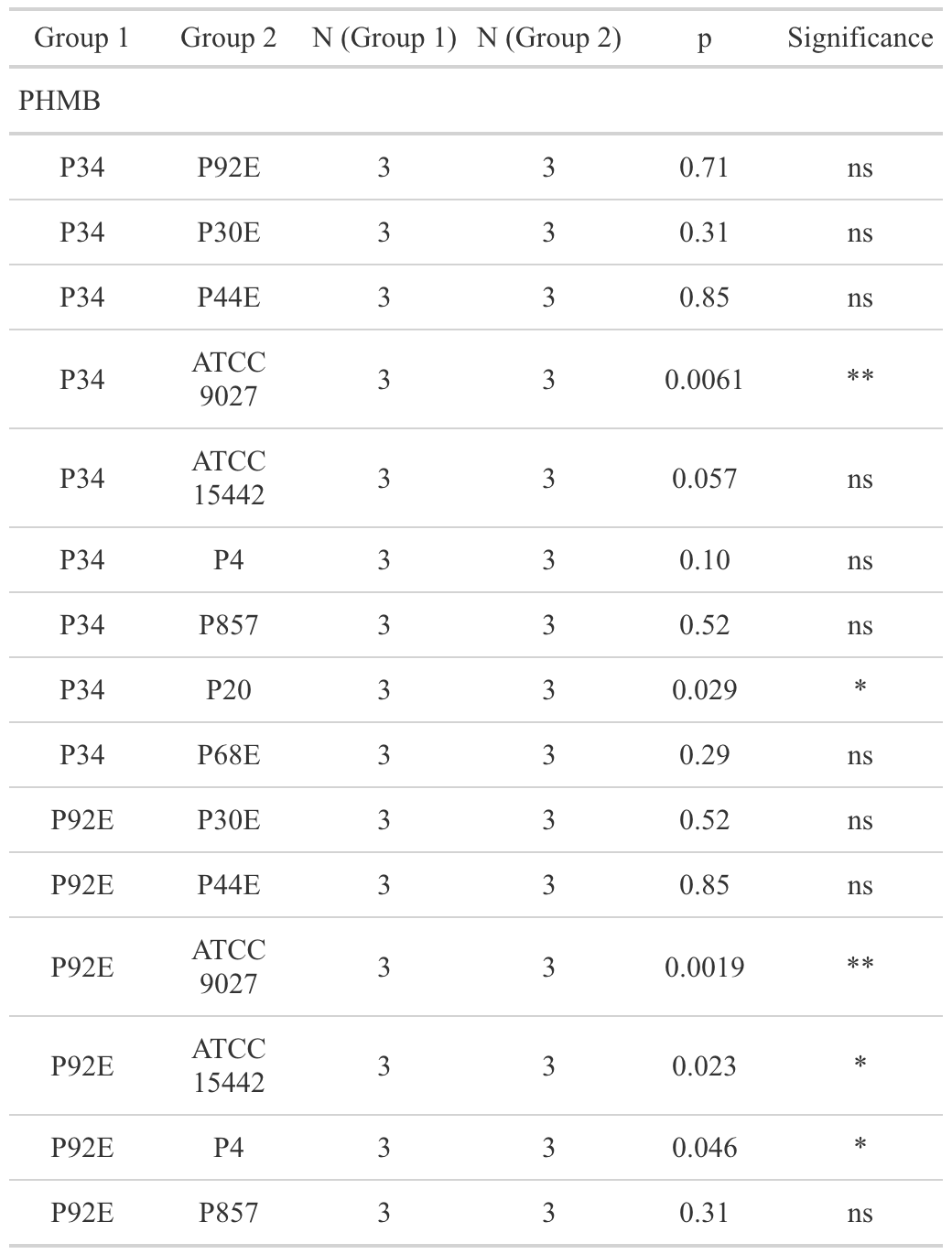


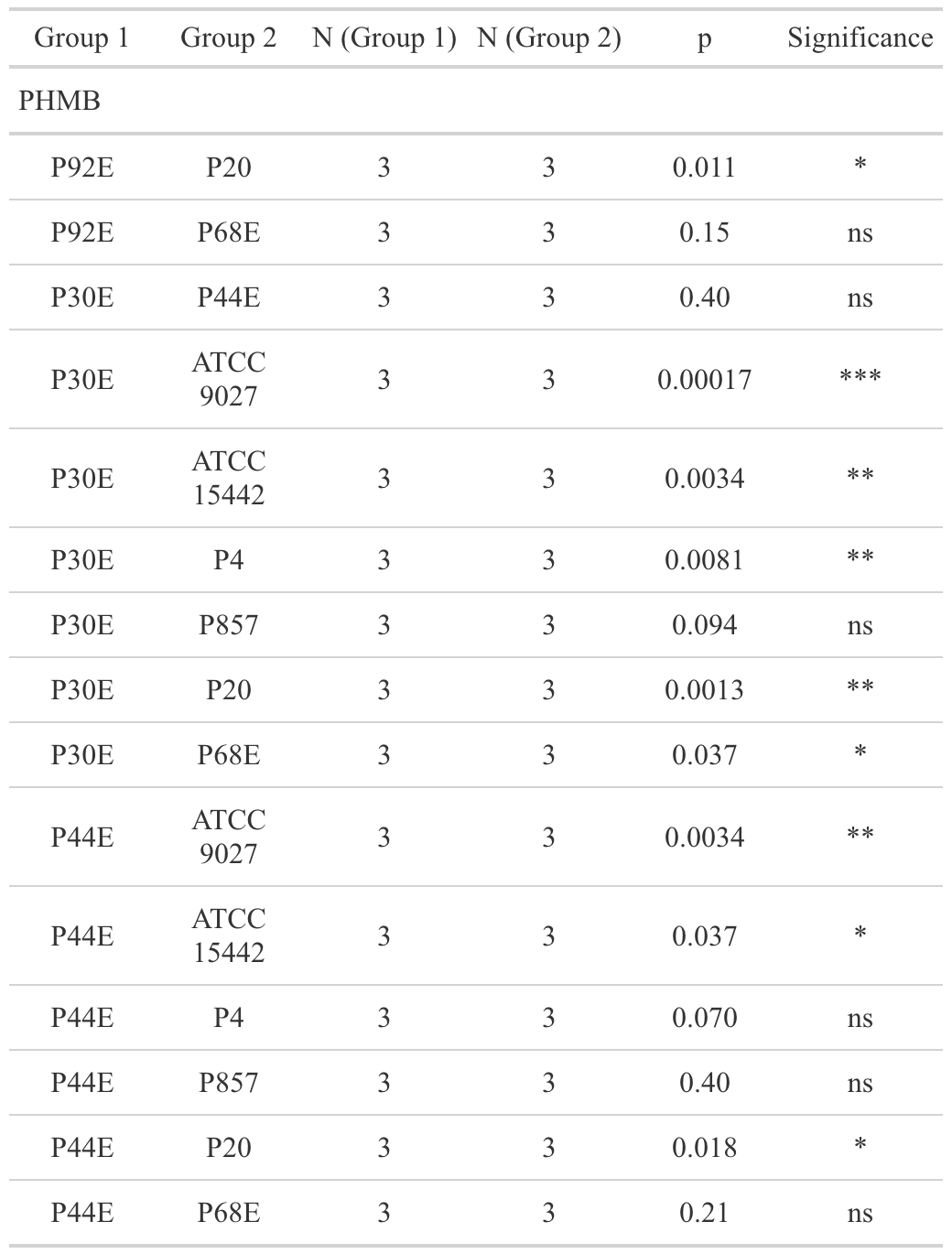


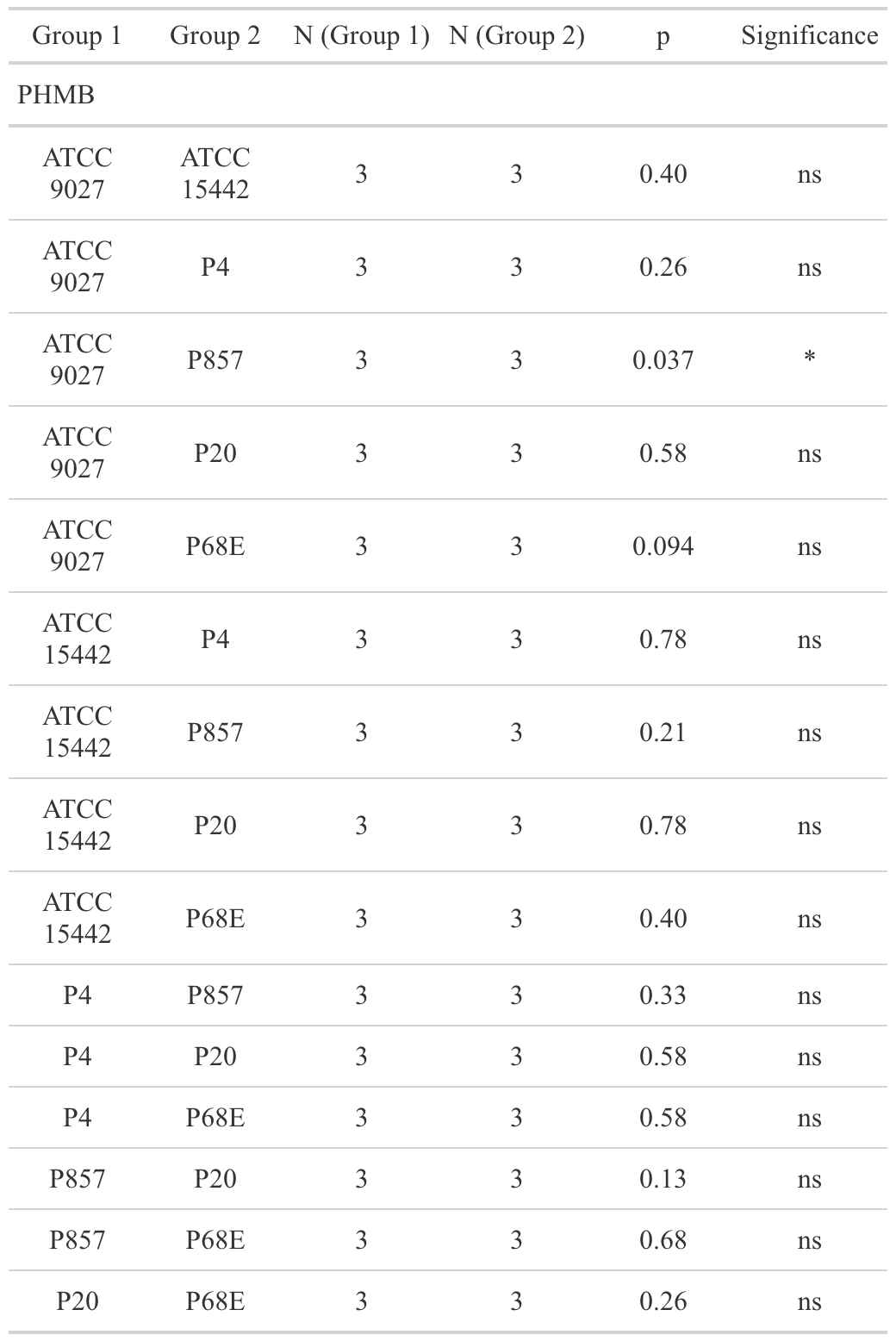


**Supplementary Table S9.** Parameters of the tested statistic of the differences between zones of growth inhibition (mm) of tested compounds against *P. aeruginosa* strains (n=10). Dunn’s test, followed by the Kruskal-Wallis test, was performed. Values of p<0.05 were considered significant. Only significant differences were included, p<=0.05 was marked with one asterisk. N- data points, TEO- Thyme Essential Oil, PHMB- polyhexanide.


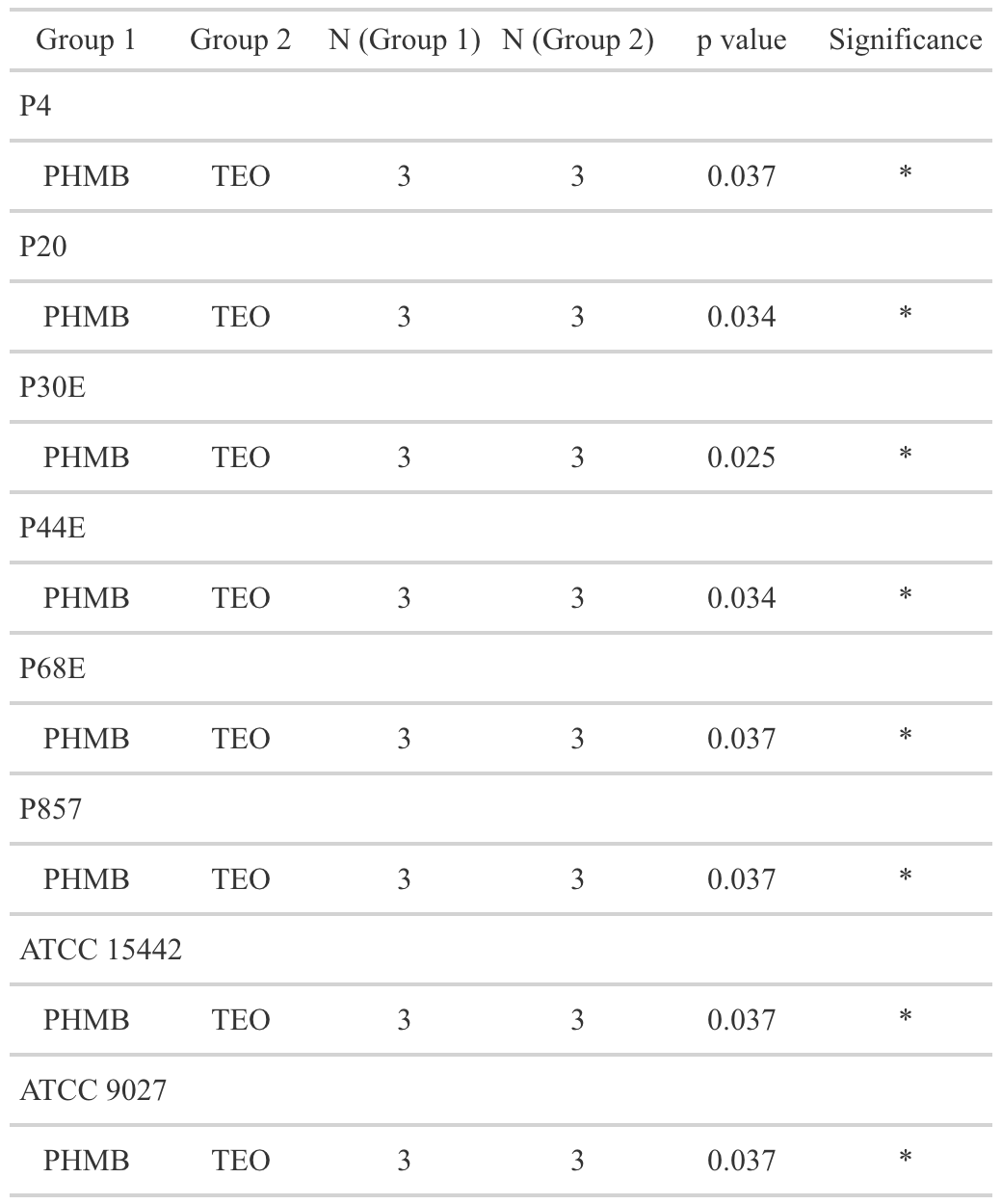


**Supplementary Table S10**. Parameters of the tested statistic of the differences between Minimal Inhibitory Concentration (MIC) (%, v/v) values of tested compounds against *P. aeruginosa* strains (n=10). Dunn’s test, followed by the Kruskal-Wallis test, was performed. Values of p<0.05 were considered significant. Only significant differences were included, p<=0.05 was marked with one asterisk. N- data points, TEO- Thyme Essential Oil, PHMB- polyhexanide.


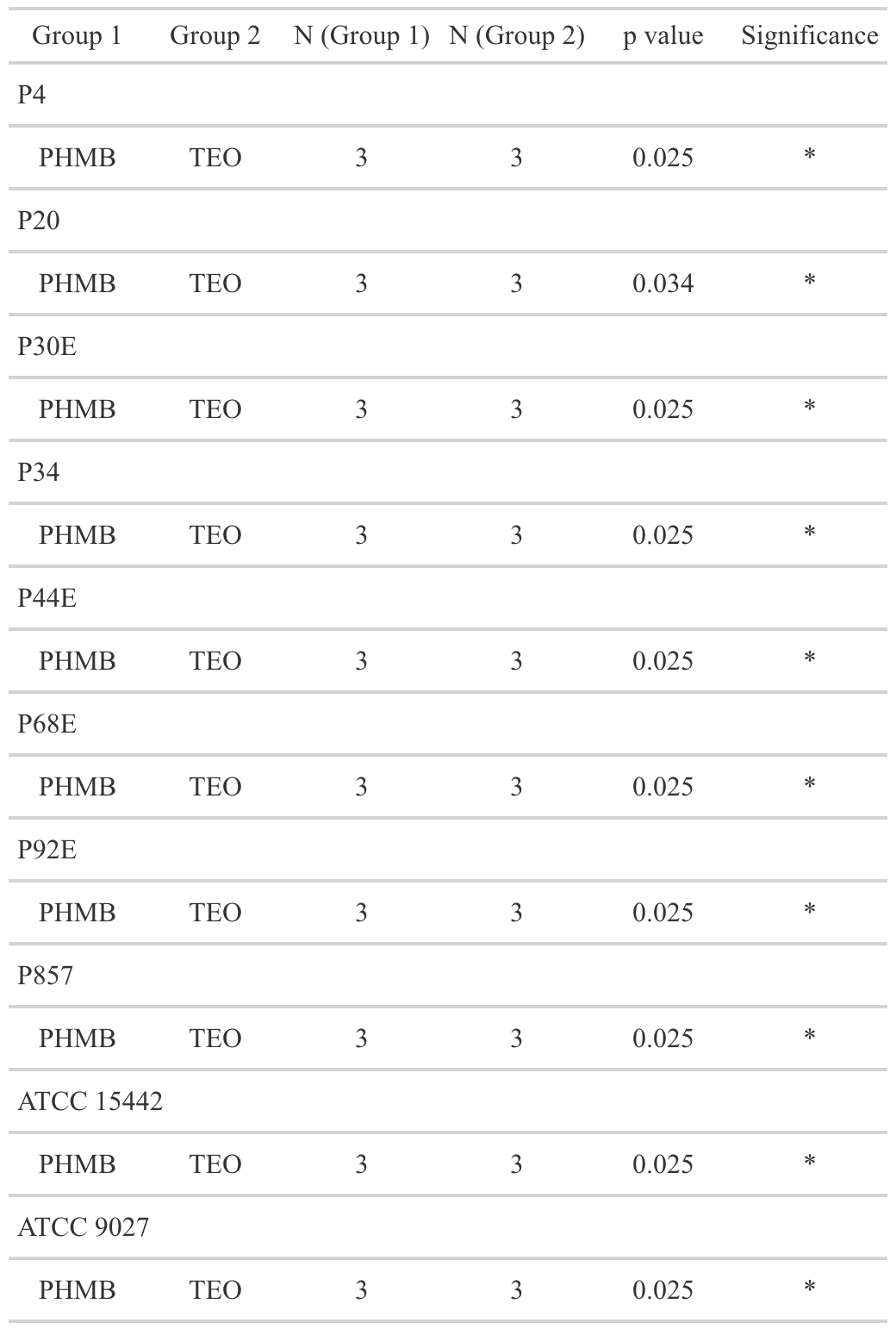


**Supplementary Table S11.** Parameters of the tested statistic of the differences between antibiofilm activity of tested compounds against *P. aeruginosa* strains (n=10). Dunn’s test, followed by the Kruskal-Wallis test, was performed. Values of p<0.05 were considered significant. Only significant differences were included, p<=0.05 was marked with one asterisk. N- data points, TEO- Thyme Essential Oil, PHMB- polyhexanide.


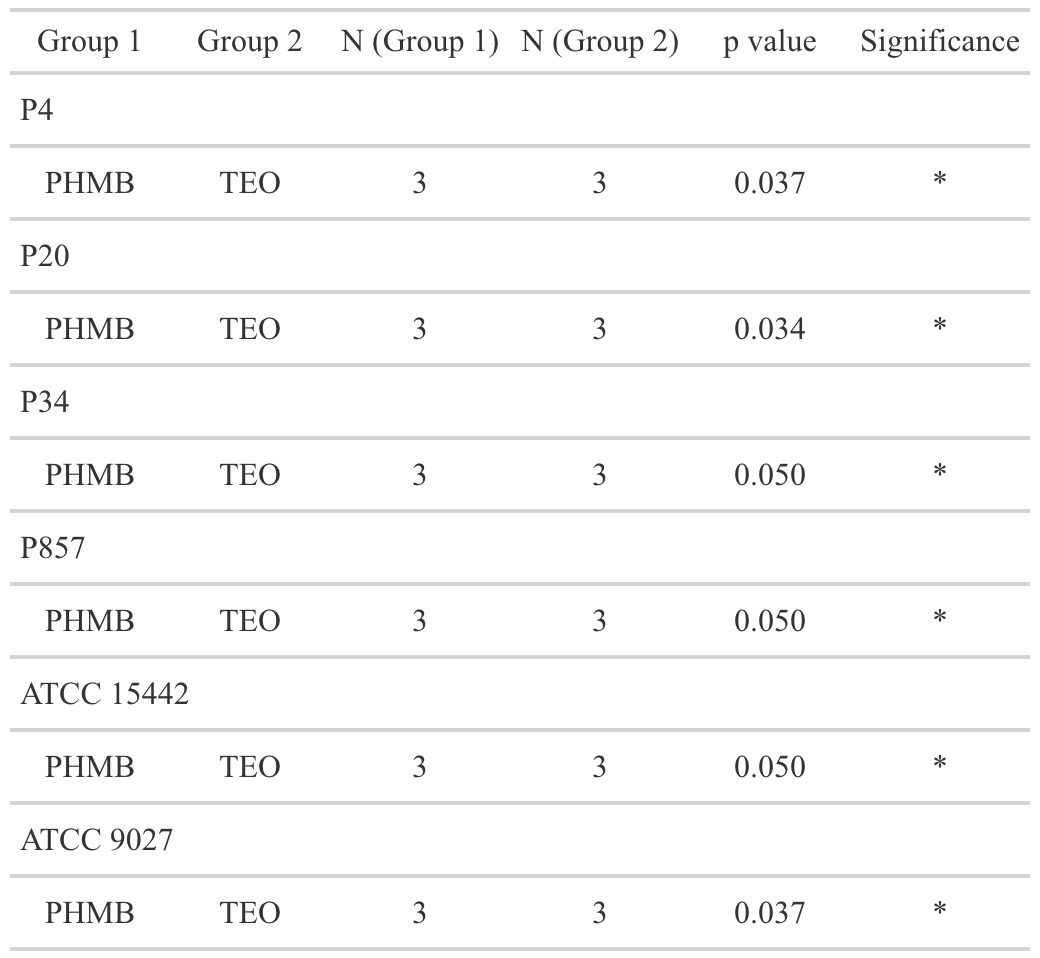


**Supplementary Table S12.** Statistical analysis of the data distribution for biofilm features across genetically distinct groups of *P. aeruginosa* strains. Distribution of biofilm mass **(A)**. Distribution of biofilm metabolic activity **(B)**. Distribution of biofilm Colony-Forming Unit number (CFU/mL) **(C)**. Normal distribution was considered for values of p>0.05 (Shapiro-Wilk test). SD- standard deviation, IQR- interquartile range (IQR), N- data points, n- number of strains included in each group.


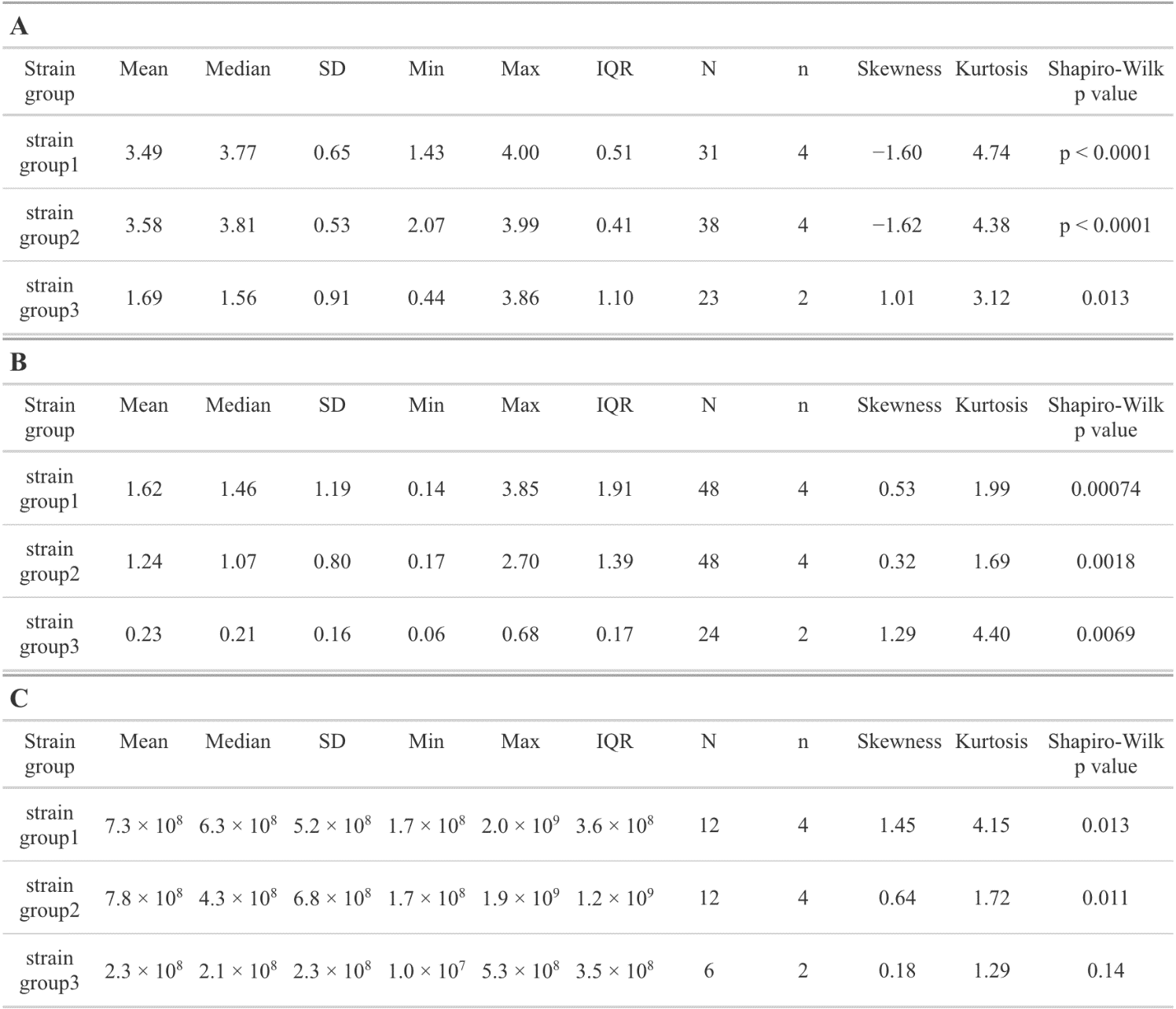


**Supplementary Table S13.** Statistical analysis of the data distribution for antimicrobial and antibiofilm activity of tested compounds against genetically distinct groups of *P. aeruginosa* strains. Growth inhibition zones values (mm) **(A)**. Minimal Inhibitory Concentration (MIC) (%, v/v) **(B)**. Biofilm cells reduction (%) **(C)**. Normal distribution was considered for values of p>0.05 (Shapiro-Wilk test). SD- standard deviation, IQR- interquartile range (IQR), N- data points, n- number of strains included in each group,NaN/ NA- not applicable. TEO- Thyme Essential Oil, PHMB- polyhexanide C+- control with Phosphate-Buffered Saline.


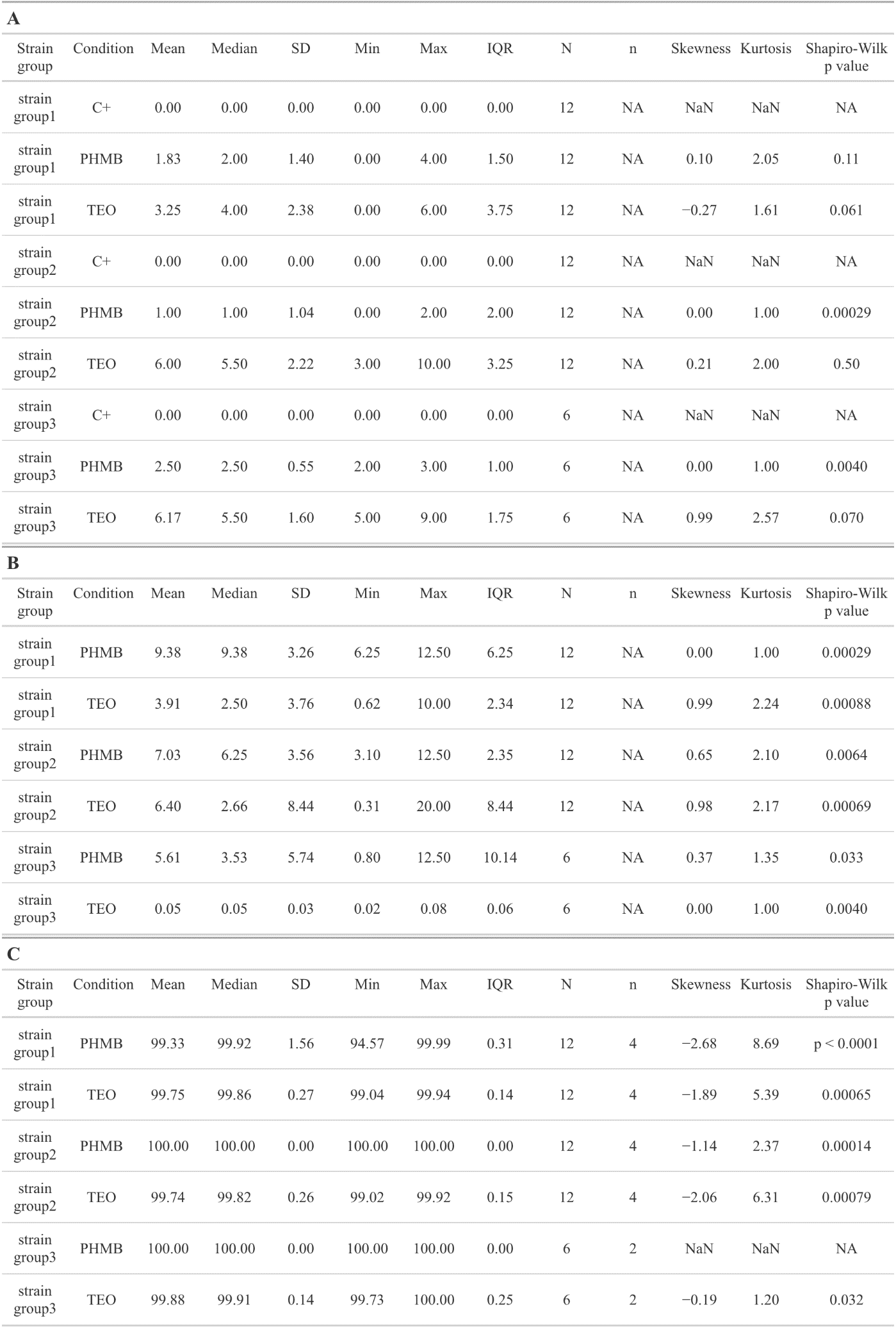


**Supplementary Table S14.** Statistic data of differences in biofilm features between *P. aeruginosa* genetically distinct groups. Biofilm mass **(A)**. Biofilm metabolic activity **(B)**. Values of p<0.05 were considered significant (Dunn’s test), p<=0.0001 was marked with four asterisks. Ns- no significant differences, N- data points, n- number of strains included in each group. For biofilm Colony-Forming Unit number (CFU/mL) p values between all groups were no significant.


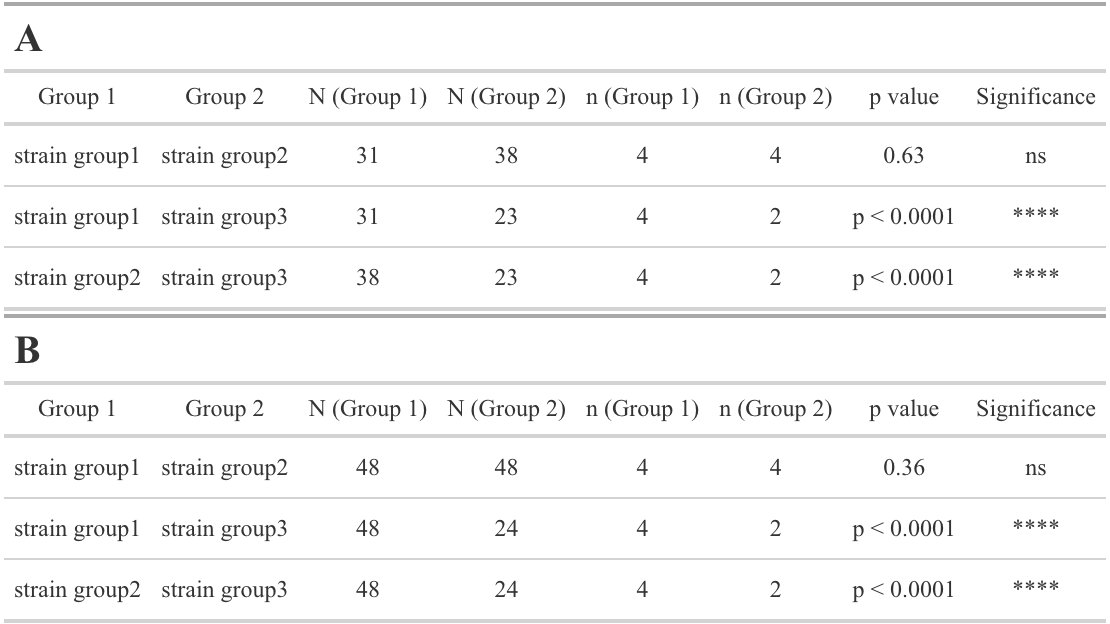


**Supplementary Table S15.** Statistic data of differences for antimicrobial and antibiofilm activity of tested compounds against genetically distinct groups of *P. aeruginosa* strains. Inhibition zone values (mm) **(A)**. Minimal Inhibitory Concentration (MIC) (%, v/v) **(B)**. Biofilm cells reduction (%) **(C).** Values of p<0.05 were considered significant (Dunn’s test), p<=0.05 was marked with one asterisk, p<=0.01 was marked with two asterisks, p<=0.001 was marked with three asterisks, and p<=0.0001 was marked with four asterisks. Ns- no significant differences, N- data points, n- number of strains included in each group. TEO- Thyme Essential Oil, PHMB- polyhexanide.


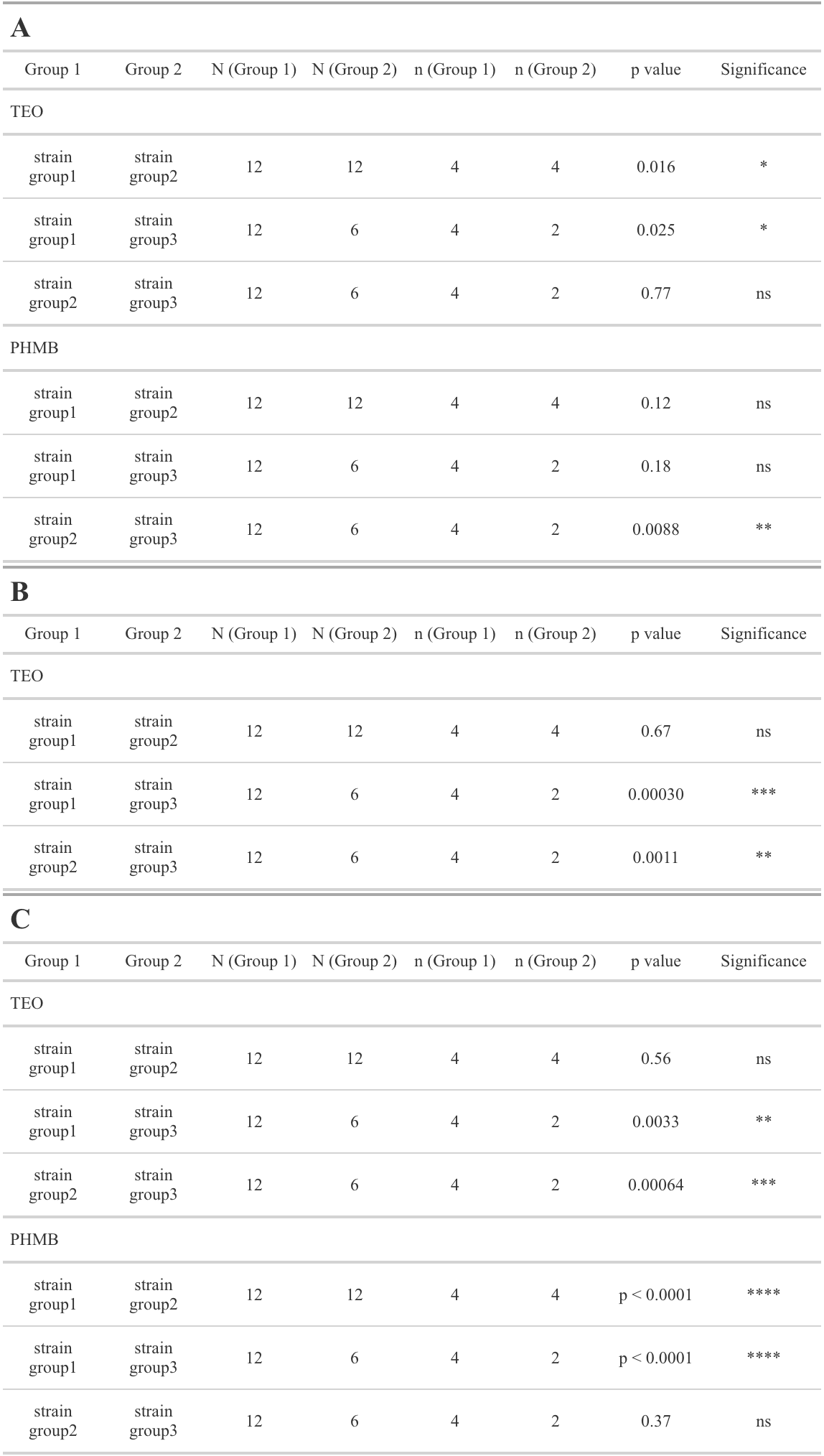

Supplement: Supplementary file 1 [file Supplementaryfile1.docx]
